# Cyclodextrin-Based Systems of *Cetraria islandica* Extracts: A Novel Approach to Improve Solubility and Biological Activity of Lichen-Derived Natural Products

**DOI:** 10.3390/molecules30153182

**Published:** 2025-07-29

**Authors:** Elżbieta Studzińska-Sroka, Karolina Cichoracka, Natalia Rosiak, Andrzej Miklaszewski, Marcin Szymański, Judyta Cielecka-Piontek

**Affiliations:** 1 Department of Pharmacognosy and Biomaterials, Poznan University of Medical Sciences, Rokietnicka 3 Str., 60-806 Poznan, Poland; karolina.pesta99@gmail.com (K.C.); nrosiak@ump.edu.pl (N.R.); jpiontek@ump.edu.pl (J.C.-P.); 2 Faculty of Materials Engineering and Technical Physics, Institute of Materials Science and Engineering, Poznan University of Technology, 60-965 Poznan, Poland; andrzej.miklaszewski@put.poznan.pl; 3 Center for Advanced Technologies, Adam Mickiewicz University in Poznań, Uniwersytetu Poznańskiego 10 Str., 61-614 Poznan, Poland; marcin.szymanski@amu.edu.pl

**Keywords:** Iceland lichen, cyclodextrin carriers, lichen extracts, biological activity, fumarprotocetraric acid

## Abstract

*Cetraria islandica* (L.) Ach. (CI) is a lichen from the Parmeliaceaea family used in medicine. However, the low solubility of CI secondary metabolites in water limits the application of lichen extract and compounds. It prompted us to study the systems of cyclodextrins (CDs) (β-CD, γ-CD, HP-β-CD, and HP-γ-CD) with the CI acetone or CI methanol extracts prepared using grinding and solvent evaporation methods. The content of fumarprotocetraric acid (FPCA), a key CI metabolite, was quantified using HPLC. CD–extract systems were characterized by X-ray powder diffraction (XRPD) and Fourier-transform infrared (FTIR) spectroscopy. Biological activity was evaluated using cell-free assays: a Folin–Ciocalteu analysis, DPPH test, acetylcholinesterase, butyrylcholinesterase, and tyrosinase inhibitions. Dissolution profiles were also assessed. The best biological and physicochemical results were obtained for systems prepared with HP-β-CD and HP-γ-CD via solvent evaporation, showing higher activity and enhanced FPCA release compared to the pure extracts. To the best of our knowledge, this is the first study to report the preparation and characterization of CD-based systems with CI extracts. The obtained results encourage us to continue our research on CI to improve the physicochemical properties of its active compounds.

## 1. Introduction

**Figure 1:**
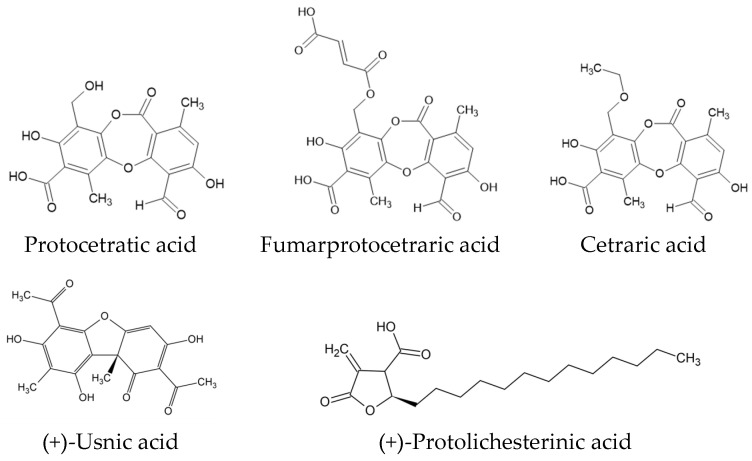
The chemical structures of the main secondary metabolites of *Cetraria islandica*.

Lichens are cosmopolitan, autotrophic organisms whose existence depends on a symbiotic relationship between fungal cells and algae or cyanobacteria. These organisms are capable of producing natural chemical compounds with unique biological properties not found in other sources. Among the many lichen species, only a few are used in traditional medicine, with *Cetraria islandica* (L.) Ach. thalli being one of the few applied in official medicine in European countries. Aqueous extracts of this species, rich in polysaccharides, are used to treat dry cough and irritation of the upper respiratory tract. Clinical studies have confirmed their efficacy in this regard [1]. In addition to polysaccharides (25–50%), the thallus of *C. islandica* is also a source of lichen-specific secondary metabolites from the depsidone group—fumarprotocetraric acid (FPCA) (2.6–11.5%), protocetraric acid (PCA) (0.2–0.3%), and trace amounts of cetraric acid [2]. This lichen also produces the aliphatic α-methylene-γ-lactone (+)-protolichesterinic acid (0.1–0.5%) (Figure 1) [3]. Some reports also indicate the presence of minor amounts of usnic acid in the raw material [4]. Moreover, *C. islandica* contains naphthoquinones (e.g., naphthazarin) [5], fatty acids (linoleic, oleic, and linolenic), carotenoids, and mineral compounds such as iodine and boron salts [6]. Studies have shown that extracts prepared with organic solvents and individual compounds from *C. islandica* possess various biological activities. In vitro tests revealed antioxidant, immunomodulatory, anti-inflammatory, antiproliferative, and cytotoxic effects. In vivo, galactomannan from water extracts showed immunomodulatory effects, while isolichenin (a polysaccharide) may enhance cognitive abilities in rodents [7]. Recent research also suggests neuroprotective potential. Fernández-Moriano et al. (2015) demonstrated that methanol extracts of *C. islandica* protect astrocytes from hydrogen-peroxide-induced damage, significantly reducing reactive oxygen species levels [8]. Other studies have shown the cytoprotective effects of FPCA on SH-SY5Y (neurons) and U373-MG (astrocytes) cell lines [9].

Despite these promising findings, the application of *C. islandica* secondary metabolites remains limited. On the one side, the lipophilic nature of some lichen compounds facilitates membrane penetration, but on the other, the lichen molecules have poor solubility in the polar environment, which decreases their bioavailability, e.g., in the digestive system [10].

Cyclodextrins (CDs) are widely used in pharmaceutical and nutraceutical formulations as functional excipients that enhance the solubility, stability, and bioavailability of lipophilic compounds. Among the natural CDs, β-CD and γ-CD are most commonly applied due to their differing cavity sizes. γ-CD, with its larger internal cavity, is particularly suitable for encapsulating bulkier or branched molecules. Moreover, γ-CD has been granted GRAS (Generally Recognized As Safe) status and is approved for use in food applications, which underscores its relevance in oral delivery systems of bioactive compounds [11]. β-CD has demonstrated effectiveness in improving both physicochemical and biological properties of plant-derived polyphenols. For example, the encapsulation of *Hypericum perforatum* extract in β-CD enhanced the thermal stability of antioxidants such as quercetin and catechins, achieving encapsulation efficiencies up to 35% [12]. Similarly, β-CD complexes of olive leaf extract improved oxidative stability in lipid-based food systems [13]. These findings highlight the potential of β-CD to protect sensitive bioactive compounds from degradation during processing and storage. Hydroxypropylated derivatives, including HP-β-CD and HP-γ-CD, offer additional advantages such as increased aqueous solubility and reduced toxicity compared to their parent CDs. HP-β-CD has been reported to enhance both the antioxidant stability and antimicrobial activity of encapsulated essential oils, such as guava leaf oil [14]. HP-γ-CD, although less frequently studied, combines the larger cavity size of γ-CD with the improved solubility and safety profile of hydroxypropylation, making it particularly suitable for poorly soluble or sensitive bioactives. Its application in functional food systems and compatibility with green processing technologies have been emphasized in recent reviews [15].

Few studies have explored the application of CD systems to improve the physicochemical properties of lichen-derived substances. One such example involves usnic acid, where CD complexation improved its solubility fivefold [16]. Kristmundsdóttir et al. (2005) showed that FPCA isolated from *C. islandica* exhibited significantly increased solubility when combined with a 10% HP-β-CD solution [17].

In light of these findings and the limited research available, we aimed to investigate the use of CD systems with *C. islandica* extracts. Specifically, we studied systems of β-cyclodextrin (β-CD), γ-cyclodextrin (γ-CD), HP-β-CD, and hydroxypropyl-γ-cyclodextrin (HP-γ-CD), with *C. islandica* acetone or methanol extracts, prepared via grinding (G) and solvent evaporation (SE) methods. Our focus was to evaluate improvements in the biological potential and release rate of FPCA, one of the main compounds in *C. islandica* thalli.

## 2. Results and Discussion

### 2.1. Phytochemical Analysis of C. islandica Extracts

#### 2.1.1. High-Performance Liquid Chromatography (HPLC) and Total Polyphenol Content (TPC) Analysis

**Figure 2:**
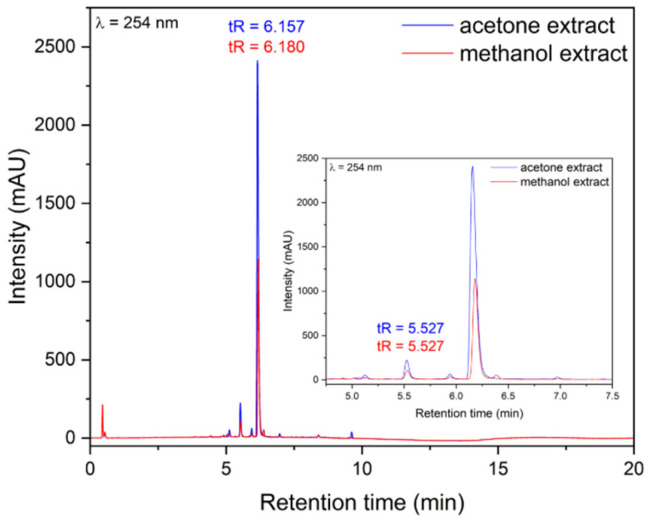
Chromatograms of *Cetraria islandica* acetone extract (blue) and methanol extract (red). Fumarprotocetraric acid was identified as the main compound in both extracts, with retention times of tR = 6.157 min for the acetone extract and tR = 6.180 min for the methanol extract; protocetraric acid was detected in smaller amounts (tR = 5.527 min).

The content of FPCA in the prepared extracts was evaluated using an HPLC analysis. The results demonstrated that both acetone extract (AE) and methanol extract (ME) obtained from *C. islandica* are rich in FPCA, with contents of 264.41 ± 0.58 mg FPCA/g of AE and 126.97 ± 1.03 mg FPCA/g of ME, respectively (Figure 2). Moreover, the presence of protocetraric acid was determined. In the acetone extract, the content of PCA was 20.60 ± 0.20 mg PCA/g AE, while 9.49 ± 0.7 mg PCA/g AE was found in the methanol extract. Additionally, the Total Polyphenol Content (TPC) was determined using the Folin–Ciocalteu method. The TPC value for the AE was 39.99 ± 1.96 mg GAE/g of extract, while for the ME, it was 7.04 ± 0.11 mg gallic acid equivalents per gram (GAE/g) of extract. Previous phytochemical studies on *C. islandica* have also confirmed the presence of FPCA in AE and ME [7,18]. Our phytochemical analysis aligns with these findings. Tas et al. (2017), in a similar study, reported that the AE of *C. islandica* is a better source of FPCA compared to the ME, with contents of 233.18 ± 0.93 mg/g and 151.64 ± 2.24 mg/g of dry extract, respectively [19]. The presence of protocetraric acid in *C. islandica* has been mentioned by many researchers, who also indicated that this compound, alongside FPCA, is an important bioactive secondary metabolite of *C. islandica* [3]. Moreover, TPC values for AEs of *C. islandica* collected from natural habitats in Poland were found to be similar to those obtained in our study, ranging between 30 mg GAE/g and 45 mg GAE/g of extract [20].

#### 2.1.2. Fourier Transform Infrared Spectroscopy with Attenuated Total Reflectance (FTIR-ATR)

**Figure 3:**
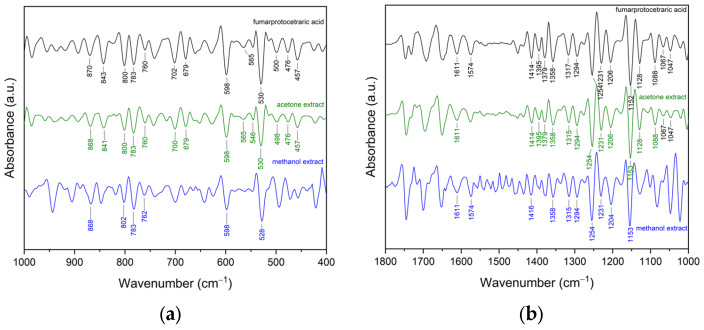
FTIR spectra of fumarprotocetraric acid, acetone extract, and methanol extract: (**a**) spectrum in the wavenumber range 1000–400 cm^−1^; (**b**) spectrum in the wavenumber range 1800–1000 cm^−1^.

The second derivative of an FTIR spectrum is commonly used to enhance spectral resolution, allowing the detection of hidden or overlapping bands. This technique highlights subtle changes in absorbance that may not be visible in the original spectrum, facilitating better identification and differentiation of functional groups or chemical environments. As demonstrated in our previous works [21,22], the application of the second derivative of FTIR spectra enabled us to confirm the presence of the main active compounds in the extracts. In this study, the presence of FPCA in the AE and ME was confirmed using the Savitzky–Golay polynomial fitting method (Figure 3a,b).

The second derivative spectra allow for the precise assignment of bands originating from the active compound in the extract, which is a matrix composed of many compounds. The peaks in the second derivative spectra of the extracts present similar absorption characteristics to the standards (Figure 3a,b). The most characteristic peaks of FPCA, along with the corresponding peaks observed in the extracts, are listed in Table S1 (Supplementary Materials). Based on this information, it can be indicated that there is more FPCA in the AE than in ME (as evidenced by the greater number of peaks originating from the standard). This is consistent with the results of the HPLC analysis, which confirmed that FPCA is more abundant in AE than in ME.

#### 2.1.3. GC-MS Analysis of the *C. islandica* Extracts

The volatile and semi-volatile compounds of *C. islandica* extracts were analyzed using GC–MS, and their compositions are presented in Table 1. The analysis was performed on both acetone (AE) and methanol (ME) extracts. A total of 19 different compounds were identified, including nine in the AE, with 8-hexadecenal, 14-methyl-, (Z)- as the major component (64.16% of total detected volatiles), and ten in the ME, with linoleic acid as the predominant compound (32.88%). However, according to the literature, volatile compounds detectable by GC–MS typically constitute less than 1% to several percent (up to 5%) of the total extract mass. The majority of extractable compounds are non-volatile [23,24].

### 2.2. Preparation and Identification of C. islandica Extract/CD Systems

The extract–cyclodextrin systems were prepared using an extract-to-cyclodextrin ratio of 1:1.5 (*w*/*w*). This proportion was specifically chosen to ensure an excess of cyclodextrin in the mixture, thereby facilitating the inclusion of bioactive components present in the extract. Similar mass-based ratios have been effectively applied in co-precipitation studies; for instance, the nanoencapsulation of thyme essential oil employed β-CD:TEO mass ratios of 8.5:1.5 and 8.5:3.0 (*w*/*w*), resulting in enhanced solubility and stability [25]. Likewise, the inclusion complexation of bacuri seed hexanic extract with β-CD demonstrated optimal gastroprotective activity at extract:CD mass ratios of 3:7 (*w*/*w*) [26]. 

Since X-ray powder diffraction (XRPD) and Fourier transform infrared spectroscopy (FTIR) are techniques used in the analysis of interactions in CD systems [22,23], both techniques were applied in our study to characterize the lichen extract/CD systems.

#### 2.2.1. X-Ray Powder Diffraction (XRPD)

**Figure 4:**
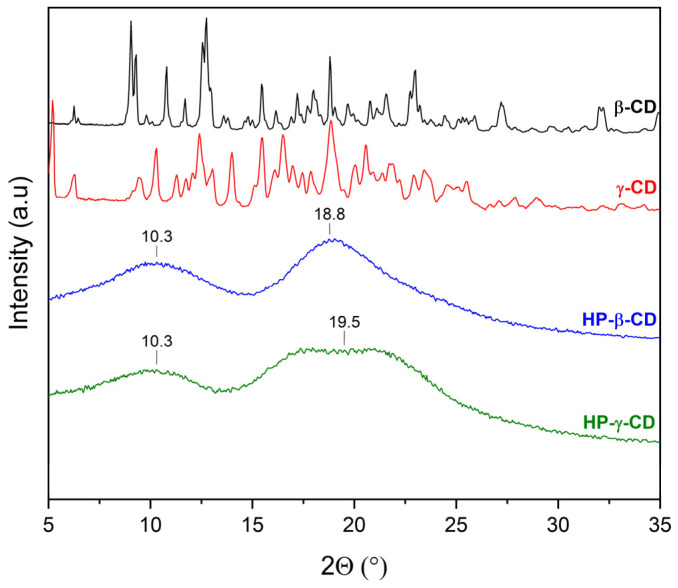
XRPD diffractograms of cyclodextrins: β-CD (black line), γ-CD (red line), HP-β-CD (blue line), and HP-γ-CD (green line).

XRPD was used to detect the complexation of CDs with extracts. The diffractograms of β-CD and γ-CD consist of numerous Bragg peaks in the 5° 2θ–35° 2θ range and clearly indicate their crystalline nature (Figure 4, black and red lines, respectively). In the case of HP-β-CD and HP-γ-CD, the presence of two broad peaks at about 10.3° 2θ and 18.8° 2θ (HP-β-CD), and 10.3° 2θ and 19.5° 2θ (HP-γ-CD), known as the halo effect, confirms their amorphous character (Figure 4, blue and green line, respectively).

**Figure 5:**
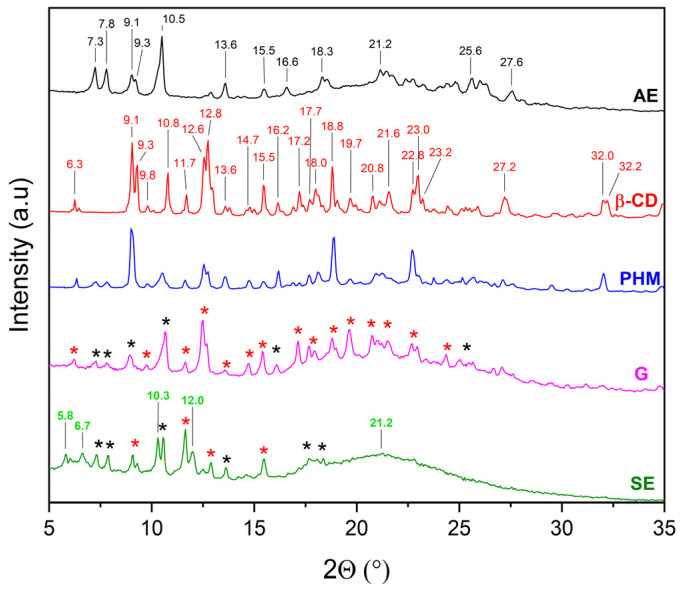
XRPD diffractograms of acetone extract (AE, black), β-cyclodextrins (β-CD, red), physical mixtures (PHMs, blue), grinding system (G, pink), and solvent evaporation system (SE, green). Bolded green peaks correspond to new Bragg peaks, while black * and red * correspond to peaks of AE and β-CD, respectively.

**Figure 6:**
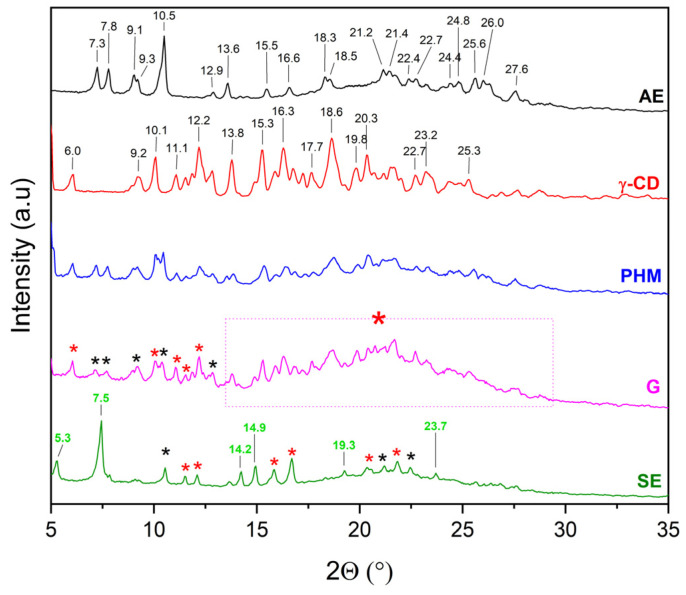
XRPD diffractograms of acetone extract (AE, black), γ-cyclodextrins (γ-CD, red), physical mixtures (PHM, blue), grinding system (G, pink), and solvent evaporation system (SE, green). Bolded green peaks correspond to new Bragg peaks, while black * and red * correspond to peaks of AE and γ-CD, respectively, pink dashed line box + red * correspond to peaks of γ-CD.

**Figure 7:**
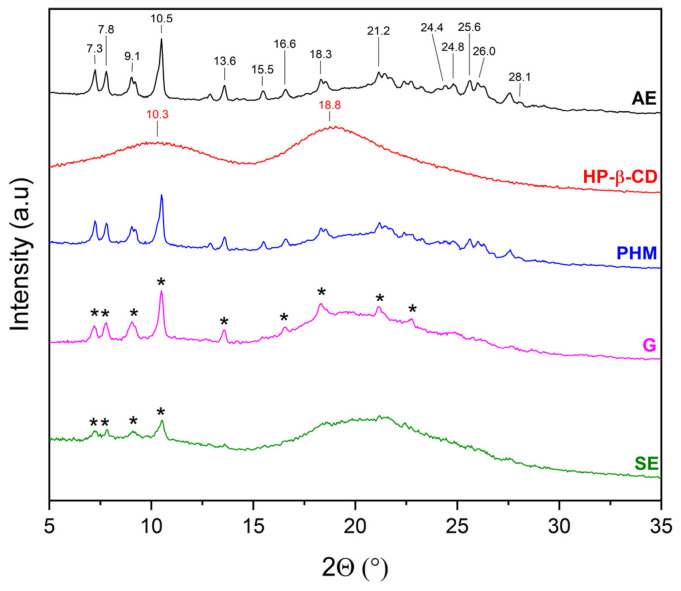
XRPD diffractograms of acetone extract (AE, black), HP-β-cyclodextrins (HP-β-CD, red), physical mixtures (PHMs, blue), grinding system (G, pink), and solvent evaporation system (SE, green). Black * corresponds to peaks of AE.

**Figure 8:**
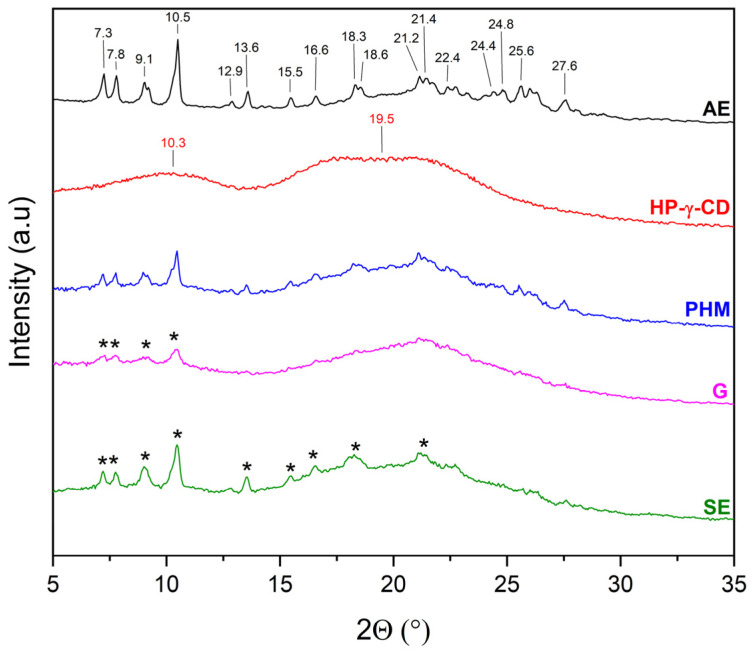
XRPD diffractograms of acetone extract (AE, black), HP-γ-cyclodextrins (HP-γ-CD, red), physical mixtures (PHMs, blue), grinding system (G, pink), and solvent evaporation system (SE, green). Black * corresponds to peaks of AE.

**Figure 9:**
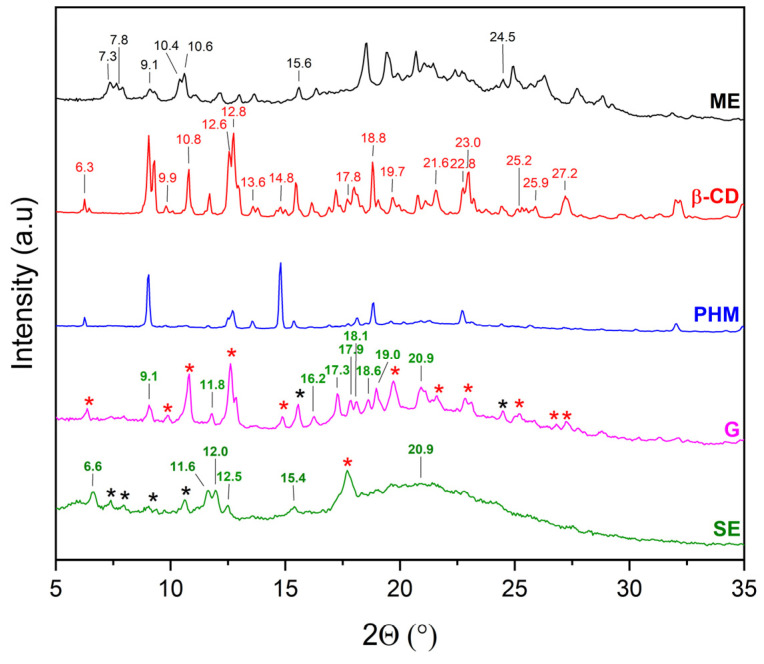
XRPD diffractograms of methanol extract (ME, black), β-cyclodextrins (β-CD, red), physical mixtures (PHM, blue), grinding systems (G, pink), and solvent evaporation systems (SE, green). Bolded green peaks correspond to new Bragg peaks, while black * and red * correspond to peaks of ME and β-CD, respectively.

**Figure 10:**
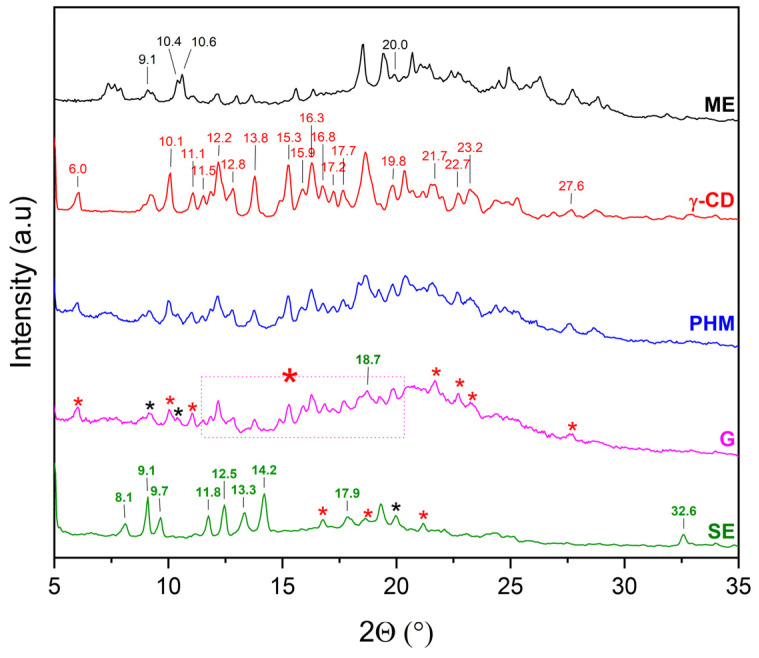
XRPD diffractograms of methanol extract (ME, black), γ-cyclodextrins (γ-CD, red), physical mixtures (PHMs, blue), grinding systems (G, pink), and solvent evaporation systems (SE, green). Bolded green peaks correspond to new Bragg peaks, while black * and red * correspond to peaks of ME and γ-CD, respectively, pink dashed line box + red * correspond to peaks of γ-CD.

**Figure 11:**
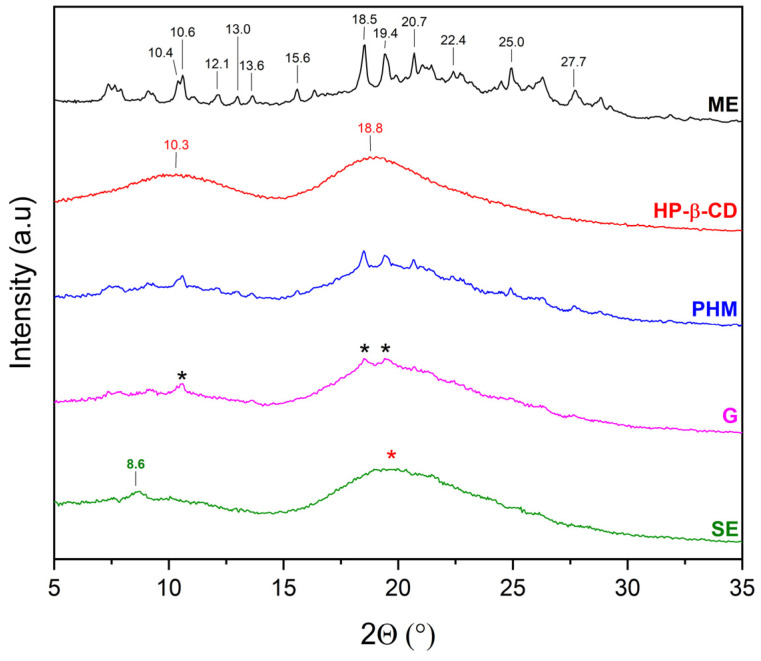
XRPD diffractograms of methanol extract (ME, black), HP-β-cyclodextrins (HP-β-CD, red), physical mixtures (PHMs, blue), grinding systems (G, pink), and solvent evaporation systems (SE, green). Bolded green peaks correspond to new Bragg peaks, while black * and red * correspond to peaks of ME and HP-β-CD, respectively.

**Figure 12:**
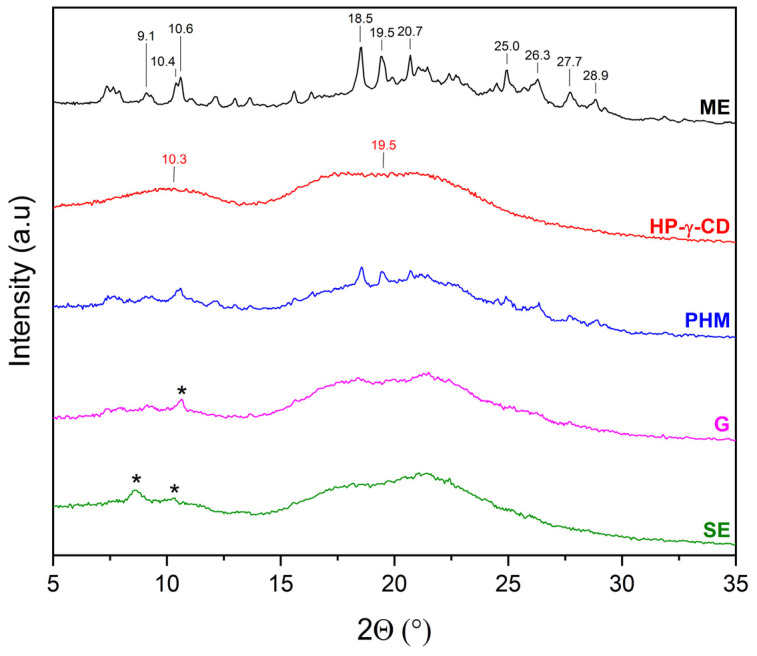
XRPD diffractograms of methanol extract (ME, black), HP-γ-cyclodextrin (HP-γ-CD, red), physical mixtures (PHMs, blue), grinding systems (G, pink), and solvent evaporation systems (SE, green). Black * corresponds to peaks of ME.

The literature [27,28,29,30,31,32] indicates that the evidence for the formation of inclusion complexes includes, among other factors, new phase formation (i.e., presence of new Bragg peaks) or not visible of characteristic Bragg peaks and the presence of a halo effect. For this reason, the diffractograms of extracts (AE and ME), CDs, AE/CD and ME/CD physical mixtures (PHMs), and AE/ME systems were compared to determine the possibility of obtaining inclusion complexes (Figure 5, Figure 6, Figure 7, Figure 8, Figure 9, Figure 10, Figure 11 and Figure 12). Diffractograms of the extract from *C. islandica* (AE) confirmed its crystalline nature, as evidenced by the presence of distinct Bragg peaks within the range of 7–28° 2θ. The physical mixtures (PHMs) of AE with various cyclodextrins (β-CD, γ-CD, HP-β-CD, and HP-γ-CD) exhibited patterns that reflected a superposition of the individual component signals (Figure 5, Figure 6, Figure 7 and Figure 8).

Comparing the diffraction patterns of AE, β-CD, and AE/β-CD (PHM) with the diffraction pattern of AE/β-CD (G), the presence of peaks originating from both AE and β-CD can be observed (Figure 5). Characteristic AE bands in the PHMs are noted, which indicates that the G method is not suitable for obtaining the AE/β-CD inclusion complex. In contrast, changes are visible in the diffraction pattern of AE/β-CD (SE). When comparing it with the diffraction patterns of AE, β-CD, and AE/β-CD (PHM), new Bragg peaks can be identified in the range of 5–12° 2θ, namely at 5.8° 2θ, 6.7° 2θ, 10.3° 2θ, and 12° 2θ. Abarca et al. [27] associate the appearance of additional Bragg peaks with the transition from cage-type to channel-type packing of β-CD. Moreover, the authors indicate in their study that such molecular structure reorganization supports the formation of inclusion complexes. In the diffraction pattern of AE/β-CD (SE), within the range of 6–19° 2θ, only a few peaks originating from AE are visible, whereas in the range of 19–30° 2θ, a broad peak with a maximum at 21.2°—characteristic of amorphous forms (the so-called halo effect)—is recorded. In the 19–30° 2θ range, the diffraction pattern of AE/β-CD (PHM) consisted of peaks originating from both AE and β-CD. The observation of a halo effect in this region may indicate partial amorphization of AE and β-CD. This is consistent with the literature [33], which indicates that the amorphous form of β-CD consists of three broad bands in the range of 5–30° 2θ, with the last band being recorded at approximately 21–30° 2θ.

Comparing the diffraction patterns of AE, γ-CD, and AE/γ-CD (PHM) with the diffraction pattern of AE/γ-CD (G), the presence of peaks originating from both AE and γ-CD can be observed (Figure 6). Characteristic AE bands in the PHM are noted, which indicates that the G method is not suitable for obtaining the AE/γ-CD inclusion complex—similarly to the case of AE/β-CD (G). As observed for AE/β-CD (SE), changes in the nature of peaks are also evident for AE/γ-CD (SE). In this case as well, new Bragg peaks can be identified, namely at 5.3° 2θ, 7.5° 2θ, 14.2° 2θ, 14.9° 2θ, 19.3° 2θ, and 23.7° 2θ. As reported by Catarino et al. [31], the appearance of additional peaks can be associated with the formation of an inclusion complex with γ-CD. The authors indicate in their study that γ-CD molecules in the inclusion complex are arranged in infinite channels. In our case, the formation of an inclusion complex contaminated with a small amount of the crystalline AE phase can be suggested (individual AE peaks observed in the range of 10–22.5° 2θ).

The diffraction pattern of HP-β-CD is characterized by two broad peaks with maxima at 10.3° and 18.8° 2θ, confirming its amorphous form [30]. In the 7–29° 2θ range, the diffraction pattern of AE/HP-β-CD (PHM) consists of peaks originating from both AE and HP-β-CD (Figure 7). Although AE/HP-β-CD (G) also contains peaks from both AE and HP-β-CD, a not visible AE peak at 15.5° 2θ and in the range of 24.0–28.1° 2θ is observed, which may indicate the partial inclusion of AE into the HP-β-CD cavity. In the case of AE/HP-β-CD (SE), peaks originating from AE were recorded only in the range of 6–11° 2θ. In the 15–30° 2θ range, a broad peak is visible, and no AE peaks are observed on its slopes—unlike in the AE/HP-β-CD (PHM) pattern, where such peaks were present in this region. This suggests that the SE method may favor the formation of inclusion complexes with HP-β-CD.

The diffraction pattern of HP-γ-CD is characterized by two broad peaks with maxima at 10.3° and 19.5° 2θ, confirming its amorphous form [34]. In the 7–29° 2θ range, the diffraction pattern of AE/HP-γ-CD (PHM) consists of peaks originating from both AE and HP-γ-CD (Figure 8). For AE/HP-γ-CD (G), AE peaks are observed only in the 6–11° 2θ range, which indicates an almost complete inclusion of AE into the HP-γ-CD cavity. In the case of AE/HP-γ-CD (SE), the same AE peaks as those recorded for AE/HP-γ-CD (PHM) are observed, suggesting that the SE method is not suitable for obtaining an inclusion complex with HP-γ-CD.

Further insight into the structural changes was provided by calculating the Crystallinity Index (CI%) of AE and CD in the prepared systems (Table 2).

The CI values of AE and CDs decreased in all systems, particularly in those prepared by the SE method, indicating a disruption of crystalline order. The most significant reductions in CI were observed for the HP-CD systems, suggesting a high degree of amorphization and complexation. The HP-β-CD:AE system showed a dramatic decrease in AE crystallinity when prepared by SE (CI: 7.74%), confirming strong interactions and successful complex formation. The HP-γ-CD:AE system, when obtained via the G method, exhibited the lowest CI (6.14%), indicating this method favored inclusion in that system.

These findings confirm that both preparation method and type of CD significantly affect the degree of crystallinity and, by extension, the formation of inclusion complexes. The SE method appears more effective for systems with HP-β-CD, while the G method better facilitates inclusion with HP-γ-CD.

The XRPD patterns of the methanolic extract from *C. islandica* (ME) confirmed its crystalline nature, as reflected by sharp Bragg reflections observed between 7° 2θ and 36° 2θ. The physical mixtures (PHMs) of ME with various cyclodextrins (β-CD, γ-CD, HP-β-CD, and HP-γ-CD) exhibited patterns that reflected a superposition of the individual component signals (Figure 9, Figure 10, Figure 11 and Figure 12).

Comparing the diffraction patterns of ME, β-CD, and ME/β-CD (PHM) with those of ME/β-CD (G) and ME/β-CD (SE), the presence of peaks originating from ME, β-CD, as well as the formation of a new phase can be identified (for G: 9.1, 11.8, 16.2, 17.3, 17.9, 18.1, 18.6, 19.0, 20.9° 2θ; and for SE: 6.6, 11.6, 12.0, 12.5, 15.4° 2θ) (Figure 9). Additionally, for SE, a broad peak with a maximum at 20.9° 2θ was recorded in the 19–30° 2θ range, which is characteristic of amorphous forms (the so-called halo effect). According to previous research [27], the appearance of additional peaks along with the observed halo effect may indicate the potential formation of a partial inclusion complex with β-CD.

Comparing the diffraction patterns of ME, γ-CD, and ME/γ-CD (PHM) with that of ME/γ-CD (G), the presence of peaks originating from both ME and γ-CD can be observed (Figure 10). Characteristic ME bands in the PHMs are noted, indicating that the G method is not suitable for obtaining the ME/γ-CD inclusion complex. Similarly to ME/β-CD (SE), changes in the nature of the peaks are observed for ME/γ-CD (SE). In this case as well, new Bragg peaks can be identified at 8.1, 9.1, 9.7, 11.8, 12.5, 13.3, 14.2, 17.9, and 32.6° 2θ. As previously reported [31], these changes may be associated with the formation of an inclusion complex with γ-CD. The presence of a single ME peak at approximately 20.0° 2θ suggests that ME/γ-CD (SE) is contaminated with a small amount of the crystalline ME phase.

The diffraction pattern of ME/HP-β-CD (PHM) consists of peaks originating from both ME and HP-β-CD, whereas the diffraction pattern of ME/HP-β-CD (G) is composed mainly of peaks characteristic of the cyclodextrin (Figure 11). Based on the recorded diffraction pattern, it can be concluded that an inclusion complex with HP-β-CD was obtained, slightly contaminated with the crystalline ME phase, as indicated by peaks at approximately 10.6°, 18.5°, and 19.4° 2θ. In the diffraction pattern of ME/HP-β-CD (SE), no characteristic ME peaks are observed. Moreover, an additional peak appears at 8.6° 2θ, and the overall shape of the pattern suggests an amorphous nature of the ME/HP-β-CD (SE) system. All of this provides evidence that the inclusion complex with HP-β-CD was successfully formed using the SE method.

The diffraction pattern of ME/HP-γ-CD (PHM) consists of peaks originating from both ME and HP-γ-CD (Figure 12). In contrast, the diffraction patterns of ME/HP-β-CD (G) and ME/HP-β-CD (SE) provide evidence for the formation of an inclusion complex with HP-β-CD, which is contaminated with a small amount of the crystalline ME phase. The formation of the inclusion complex is indicated by the absence of characteristic ME peaks observed in the PHM pattern, as well as by the presence of a characteristic broad peak in the range of 15–30° 2θ.

The diffractograms of ME/HP-β-CD and ME/HP-γ-CD systems were dominated by CD-specific peaks, with only several reflections attributable to ME. This pattern implies that the extract was largely incorporated into the CD cavities, resulting in a significant loss of its crystalline features.

Further insight into the structural changes was provided by calculating the Crystallinity Index (CI%) of ME and CD in the prepared systems (Table 3).

This interpretation is reinforced by the Crystallinity Index (CI%) results. In most systems, a significant reduction in CI was observed, particularly in those prepared by SE. In the ME/β-CD system, the CI of ME dropped from 72.32% in the raw state to 19.65% after the SE treatment, and the CI of β-CD decreased to 34.74%. Even more dramatic changes were seen in the grinding method, where the CI of β-CD fell to 2.79%, confirming a near-complete loss of its crystalline structure, although the crystallinity of ME remained relatively high (63.43%). In the ME/γ-CD system, SE also caused a notable decline in ME crystallinity (25.36%), while grinding led to a very low CI of 5.11%, indicating extensive amorphization. The most substantial reductions were observed in systems with hydroxypropylated CDs. In the ME/HP-β-CD system, SE decreased the CI of ME to 1.88%, and in the ME/HP-γ-CD system prepared by grinding, the CI dropped to 4.34%, both reflecting a nearly complete loss of crystallinity.

These findings confirm that the applied preparation methods, particularly solvent evaporation, and the type of cyclodextrin, especially hydroxypropylated derivatives, play a crucial role in modifying the crystalline properties of ME. The pronounced reduction in crystallinity and the appearance of new diffraction peaks strongly indicate the formation of inclusion complexes and the effective incorporation of ME into the CD matrices.

#### 2.2.2. Fourier Transform Infrared Spectroscopy with Attenuated Total Reflectance (FTIR-ATR)

**Figure 13:**
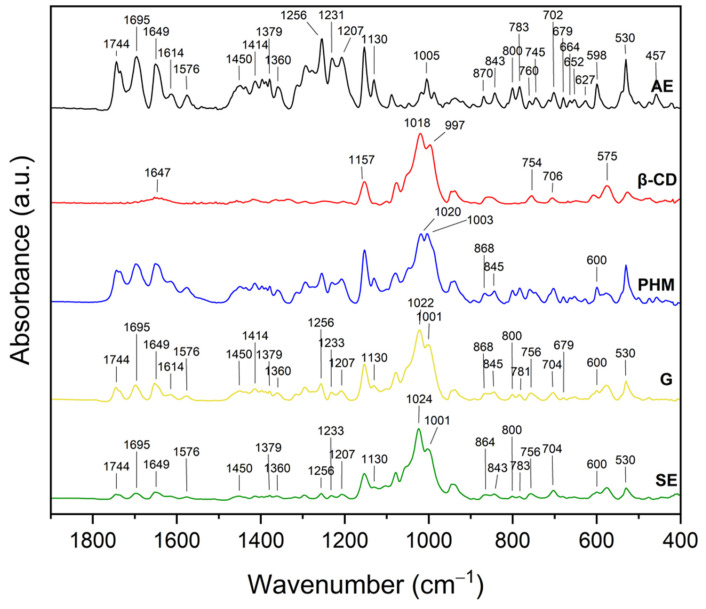
FTIR analysis: acetone extract (AE) (black), β-cyclodextrin (β-CD) (red), physical mixture (PHM) (blue), system by grinding (G) (yellow), and system by solvent evaporation (SE) (green).

**Figure 14:**
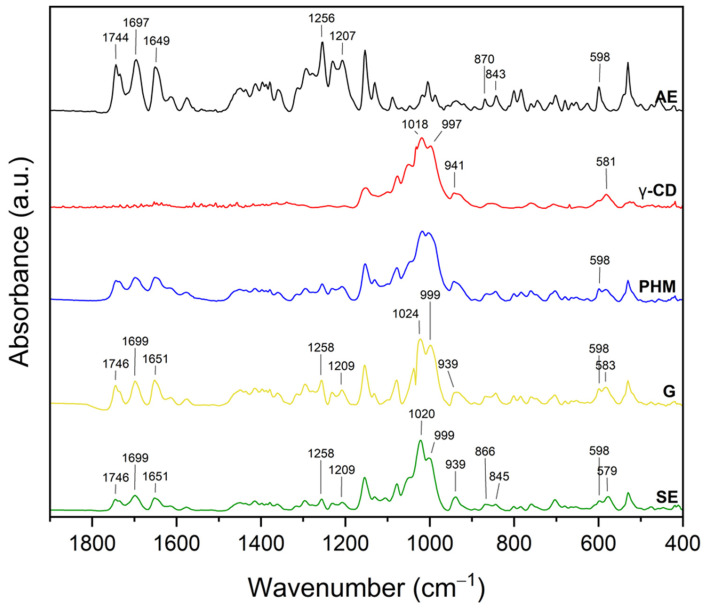
FTIR analysis: acetone extract (AE) (black), γ-cyclodextrin (γ-CD) (red), physical mixture (PHM) (blue), system by grinding (G) (yellow), and system by solvent evaporation (SE) (green).

**Figure 15:**
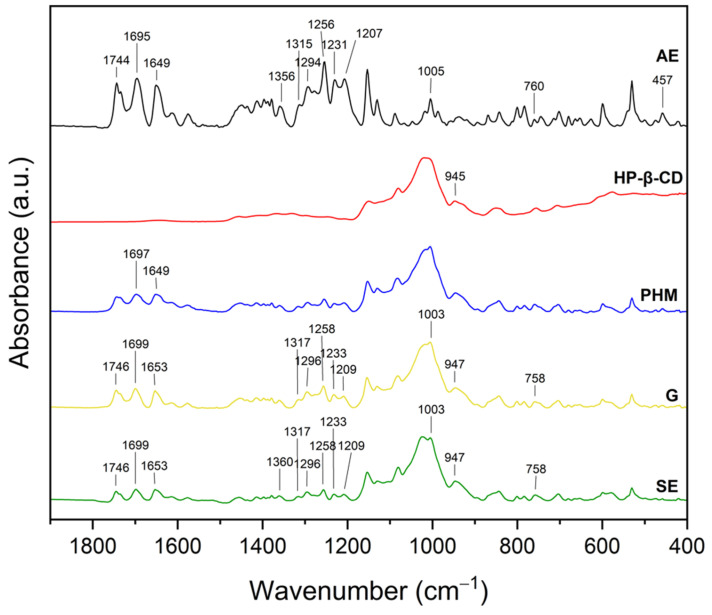
FTIR analysis: acetone extract (AE) (black), 2-hydroxypropyl-β-cyclodextrin (HP-β-CD) (red), physical mixture (PHM) (blue), system by grinding (G) (yellow), and system by solvent evaporation (SE) (green).

**Figure 16:**
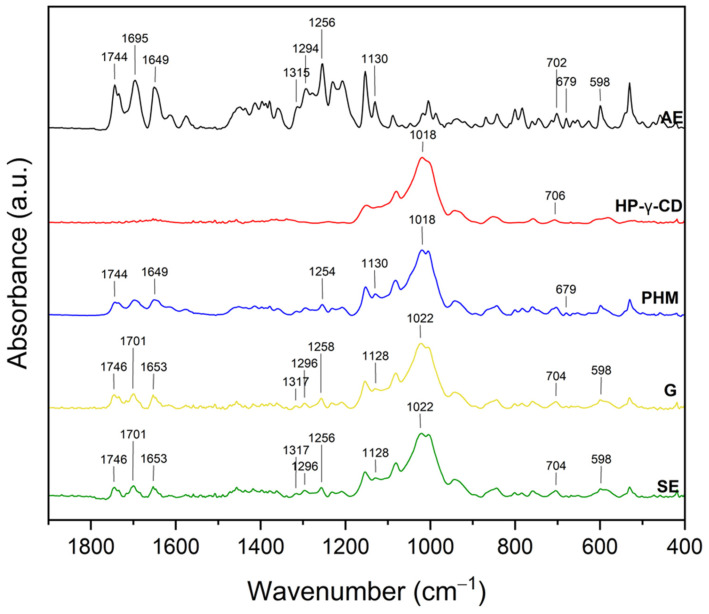
FTIR analysis: acetone extract (AE) (black), hydroxypropyl-γ-cyclodextrin (HP-γ-CD) (red), AE/HP-γ-CD 1:1.5 physical mixture (PHM) (blue), AE/HP-γ-CD 1:1.5 system by grinding (G) (yellow), and AE/HP-γ-CD 1:1.5 system by solvent evaporation (SE) (green).

**Figure 17:**
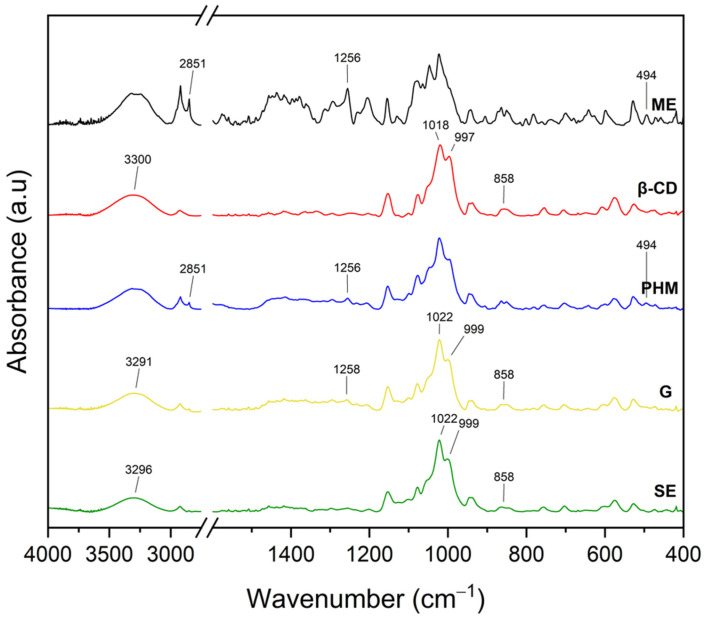
FTIR analysis: methanol extract (ME) (black), β-cyclodextrin (β-CD) (red), physical mixture (PHM) (blue), system by grinding (G) (yellow), and system by solvent evaporation (SE) (green).

**Figure 18:**
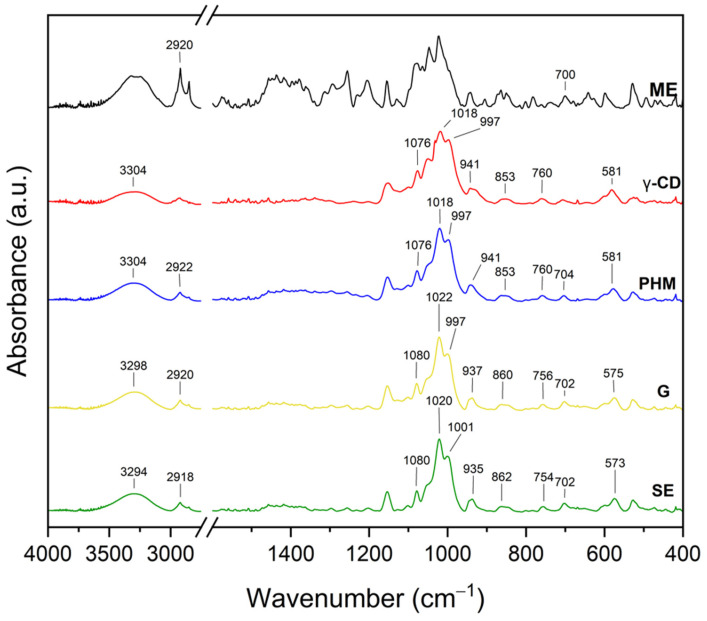
FTIR analysis: methanol extract (ME) (black), γ-cyclodextrin (γ-CD) (red), physical mixture (PHM) (blue), system by grinding (G) (yellow), and system by solvent evaporation (SE) (green).

**Figure 19:**
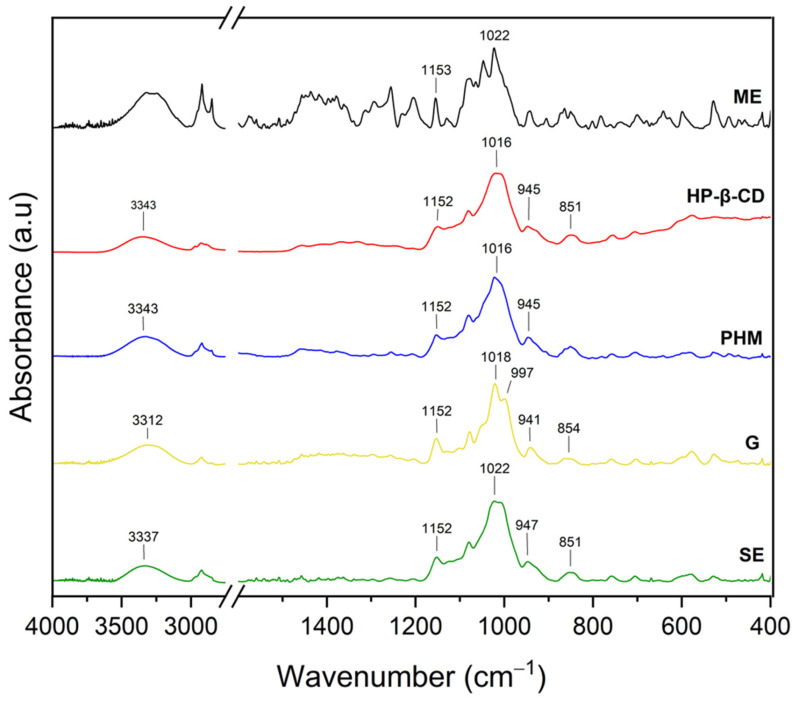
FTIR analysis: methanol extracts (MEs) (black), 2-hydroxypropyl-β-cyclodextrin (HP-β-CD) (red), physical mixture (PHM) (blue), system by grinding (G) (yellow), and system by solvent evaporation (green).

**Figure 20:**
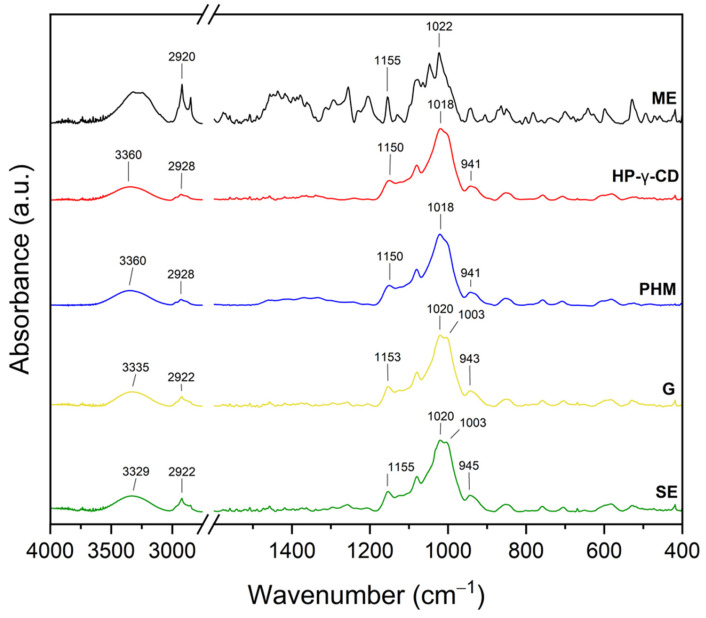
FTIR analysis: methanol extract (ME) (black), hydroxypropyl-γ-cyclodextrin (HP-γ-CD) (red), physical mixture (PHM) (blue), system by grinding (G) (yellow), and system by solvent evaporation (SE) (green).

The infrared spectra of the *C. islandica* crude extracts (AE and ME), pure CDs (β-CD, γ-CD, HP-β-CD, and HP-γ-CD), AE or ME/CD systems (with β-CD, γ-CD, HP-β-CD, and HP-γ-CD) prepared by G or by SE and PHMs, each of *C. islandica* extracts with CDs, are presented in Figure 13, Figure 14, Figure 15, Figure 16, Figure 17, Figure 18, Figure 19 and Figure 20. The FTIR spectra of the AE and ME are characterized by numerous bands in the range of 400–3000 cm^−1^. Based on the second derivative for the recorded FTIR spectra, peaks corresponding to FPCA in AE and ME were identified (Table S1).

The literature suggests that the range 2900–2950 cm^−1^ can be attributed to the asymmetric stretching vibrations of the CH_3_ group. The peaks in the range 1400–1600 cm^−1^ can indicate C=C stretching vibrations from the compounds in the extract. The peaks from 1150–1300 cm^−1^ can be attributed to the ether bond [35]. The FTIR spectrum of β-CD has characteristic bonds at 754 (O-H, C-H bending vibrations in and out of the plane), 1018 cm^−1^ (COC), 1076 cm^−1^ (stretching vibration of the C-O), 1153 cm^−1^ (wagging vibration of the C-H bonds directly at the sugar ring), 1647 cm^−1^ (H–O–H deformation bands of water present in CD), 2920 cm^−1^ (CH_2_ and CH_3_ stretching), and 3383 cm^−1^ (OH stretching) [36,37]. γ-CD has characteristic bonds at 858 cm^−1^ (C-C-H, C-O, and C-C bending from anomeric vibration), 941 cm^−1^ (skeletal vibration involving α-1,4-linkage), 1018 cm^−1^ (C-C-O stretching), 1153 cm^−1^ (C-O-C asymmetric bending), 2930 cm^−1^ (C-H asymmetric stretching), and 3304 cm^−1^ (OH stretching) [38]. HP-β-CD has characteristic bonds at 847 cm^−1^ (hydrogen bond formation between primary and secondary OH groups and the presence of glucopyranose units of HP-β-CD in C1 chair conformation), 945 cm^−1^ (the presence of glucopyranose units of HP-β-CD in C1 chair conformation), 1012 cm^−1^ (C-O-C stretching), and 3343 cm^−1^ (O-H stretching) [30].

The FTIR spectra of the systems formed between the AE and various CDs (β-CD, γ-CD, HP-β-CD, and HP-γ-CD) revealed significant changes, indicating the formation of inclusion complexes and/or molecular interactions (Figure 6, Figure 7, Figure 8 and Figure 9). Tables S2 and S5 summarize the observed changes in the spectra of pure components, PHMs, and AE/CD systems prepared by G or SE. Based on the second derivative of the recorded FTIR spectra of AE, the peaks corresponding to FPCA were identified (bolded values in Tables S2–S5).

In the AE/β-CD systems (Figure 13, Table S2), the formation of interactions is evidenced by the not visible, shifts, or intensity reductions in several characteristic AE bands [39].

Notably, not visible AE bands (due to a mass dilution effect) at 457, 530, 627, 652, 664, 679, 754, 843, 1360, 1450, and 1614 cm^−1^ suggest that the corresponding functional groups may be directly involved in host–guest interactions with the β-CD cavity. Not visible AE bands can result from the restriction of vibrational freedom due to encapsulation or from overlapping with CD signals in the same region.

Several other bands showed reduced intensity or peak shifts, including those at 598, 600, 704, 783, 845, 868, 1001, 1018–1024, and 1233 cm^−1^. For example, the band at 1018 cm^−1^, typically associated with C–O–C stretching vibrations in β-CD, shifted to 1022 cm^−1^ in the G system and to 1024 cm^−1^ in the SE system. This shift reflects perturbations in the CD skeleton caused by guest molecule inclusion. Similarly, the shift in the 754 cm^−1^ band, associated with O–H and C–H bending vibrations, to 756 cm^−1^ in both methods suggests modifications in the hydrogen bonding environment.

Additionally, the broad –OH stretching band observed at ~3300 cm^−1^ in pure β-CD shifted to 3329 cm^−1^ in the AE/β-CD complexes (Figure S1), which is indicative of altered hydrogen bonding interactions. This shift suggests the involvement of hydroxyl groups from β-CD in new hydrogen bonds with AE components, particularly FPCA derivatives that possess polar functional groups such as hydroxyl and carboxyl moieties. These interactions likely occur at the CD rim rather than deep within the hydrophobic cavity, suggesting partial inclusion and external complexation.

The presence of residual AE signals in the spectra of the inclusion complexes confirms that not all components are fully encapsulated, supporting the hypothesis of incomplete inclusion complexes. This is consistent with the XRPD results, where only a partial loss of crystallinity was observed, especially for β-CD-based systems. The hydrophobic interactions between nonpolar parts of AE (e.g., aromatic rings from FPCA derivatives) and the nonpolar cavity of the cyclodextrins may contribute to stabilizing the inclusion, while hydrogen bonds formed between hydroxyl groups of CDs and polar functionalities of AE further stabilize the supramolecular structure.

The FTIR spectra of the AE/γ-CD systems (Figure 14 and Figure S1, and Table S3), prepared by both grinding (G) and solvent evaporation (SE), showed changes indicative of intermolecular interactions, although these alterations were less pronounced than those observed in the AE/β-CD systems.

Notable shifts in band positions were detected at 581, 598, 843, 870, 997, 1018, 1207, 1256, 1649, 1695, and 2920 cm^−1^. Importantly, in contrast to the AE/β-CD systems, AE-specific bands were recorded, suggesting that inclusion was only partial, and the extent of interaction between AE and γ-CD was lower.

Further analysis revealed that bands characteristic of γ-CD itself (581, 941, 997, 1018, and 3304 cm^−1^) also shifted, with peaks moving in the G system to 583, 939, 999, and 1024 cm^−1^ and in the SE system to 579, 939, 999, 1020, and 3316 cm^−1^. These changes suggest modifications in the vibrational environment of γ-CD upon complexation, particularly in the glycosidic C–O–C and –OH groups, which may be engaged in hydrogen bonding with polar AE constituents. The shift in the broad –OH stretching band from 3304 to 3316 cm^−1^ confirms the formation of new hydrogen bonds between AE and γ-CD, although these are likely weaker or less extensive than those observed in β-CD-based systems.

These findings imply that the host–guest interactions in AE/γ-CD systems are predominantly hydrogen bonds and weaker hydrophobic effects, likely due to the larger internal cavity of γ-CD, which may not accommodate AE components as effectively. This interpretation aligns with the XRPD data, which showed a lower degree of AE incorporation into γ-CD compared to β-CD, supporting the conclusion of less stable or less complete inclusion complexes [40].

In the case of AE/HP-β-CD systems (Figure 15, Table S4), the FTIR spectra confirmed more pronounced spectral changes, indicative of stronger interactions.

Specifically, not visible AE bands (due to the mass dilution effect) at 457 and 760 cm^−1^, combined with significant shifts in bands around 945, 1005, 1256, 1294, 1315, and 1356 cm^−1^, points to a substantial disruption of the original vibrational modes of the extract. These changes likely result from the effective inclusion of AE into the HP-β-CD cavity, as well as the formation of multiple hydrogen bonds between hydroxyl groups of HP-β-CD and polar functional groups in AE [41]. Importantly, the -OH stretching vibration shifted from 3343 cm^−1^ in pure HP-β-CD to 3358 cm^−1^ in the AE/HP-β-CD system, further confirming alterations in the hydrogen bonding network. The greater shift in the hydroxyl band compared to γ-CD or β-CD systems suggests that HP-β-CD engages more strongly in hydrogen bond formation, likely due to the increased number of accessible -OH groups and enhanced solubility of the HP-modified CD [42]. This is supported by the XRPD analysis, which showed that the SE-prepared AE/HP-β-CD system exhibited nearly complete inclusion, indicative of a stable and efficient host–guest complex stabilized primarily by hydrogen bonds.

Similarly, in the AE/HP-γ-CD systems (Figure 16, Table S5), characteristic spectral changes point to the formation of inclusion complexes.

Not visible AE bands at 679 and 702 cm^−1^ were observed, along with notable shifts in bands from 1018 to 1022 cm^−1^, 1256 to 1258 cm^−1^, 1649 to 1653 cm^−1^, 1695 to 1701 cm^−1^, and 1744 to 1746 cm^−1^. These shifts primarily involve C-O, C=O, and aromatic group vibrations, suggesting that both hydrogen bonding and π–π or hydrophobic interactions may contribute to the stabilization of the inclusion complexes [43].

As in the AE/HP-β-CD system, the -OH stretching band underwent a shift to 3358 cm^−1^, confirming the formation of new hydrogen bonds between AE and HP-γ-CD [44]. However, the XRPD analysis indicated that the grinding (G) method led to a more effective inclusion in this system, suggesting that mechanical activation favored the penetration of AE into the HP-γ-CD cavity and promoted stronger host–guest interactions, especially hydrophobic ones within the expanded inner space of the γ-CD framework.

In summary, the FTIR data reveal that the type of cyclodextrin and the preparation method significantly influence the nature and strength of the molecular interactions formed between CD and AE. Hydrogen bonding dominates the interactions, particularly in systems with hydroxypropylated CDs, while hydrophobic interactions are more relevant in the encapsulation process involving native γ-CD and AE aromatic components. The complementary XRPD results reinforce the FTIR-based interpretation, confirming that the extent of crystallinity loss and band alteration correlates with the degree of inclusion and interaction strength.

The FTIR spectra of the systems formed between the methanolic extract (ME) and various cyclodextrins (CDs) revealed characteristic spectral modifications, suggesting the presence of molecular interactions and potential inclusion complex formation (Figure 17; Tables S6–S9). Notably, in contrast to the AE-based systems, most of the observed changes in the ME-based systems were associated with the bands characteristic of the CDs, rather than distinct ME bands, which may result from the lower crystallinity of the ME or a different profile of active compounds compared to AE.

In the ME/β-CD system (Figure 17, Table S6), significant changes were observed in the β-CD-associated bands.

The characteristic C-O-C stretching band at 1018 cm^−1^ shifted to 1022 cm^−1^ in both G and SE systems, while the broad -OH stretching vibration at 3300 cm^−1^ shifted downfield to 3291 cm^−1^ (G) and 3296 cm^−1^ (SE). Additionally, the ME-specific band at 494 cm^−1^ is not visible in both systems, suggesting a direct interaction or partial encapsulation of compounds responsible for that signal. The red shift in the OH region confirms the formation of hydrogen bonds between hydroxyl groups of β-CD and polar functional groups of ME, likely phenolic acids or depsides. Not visible bands (e.g., at 2851 cm^−1^) and intensity changes in other regions further support structural rearrangements due to host–guest complexation [39,45].

For ME/γ-CD systems (Figure 18, Table S7), spectral changes were also observed but were generally less pronounced.

The γ-CD band at 1018 cm^−1^ shifted to 1022 cm^−1^ (G) and 1020 cm^−1^ (SE), while the –OH stretching band at 3304 cm^−1^ shifted to 3298 cm^−1^ (G) and 3294 cm^−1^ (SE). The skeletal vibration at 941 cm^−1^ also shifted to 937 cm^−1^ (G) and 935 cm^−1^ (SE). These subtle shifts are indicative of weaker hydrogen bonding and likely the partial inclusion of ME constituents into the γ-CD cavity. The broader cavity of γ-CD may lead to a more flexible, but less tightly bound, complex. Visible ME bands suggest surface or partial interaction rather than full inclusion [40].

More distinct spectral changes were noted in the ME/HP-β-CD system (Figure 19, Table S8).

The band at 945 cm^−1^, associated with the C1 chair conformation of glucopyranose units in HP-β-CD, shifted to 941 cm^−1^ (G) and 947 cm^−1^ (SE), while the band at 851 cm^−1^ (the hydrogen bond network in HP-β-CD) remained mostly stable. The O-H stretching band experienced a marked shift from 3343 cm^−1^ in HP-β-CD to 3312 cm^−1^ (G) and 3337 cm^−1^ (SE), confirming the establishment of new hydrogen bonds between ME and CD. The stronger redshift in the G system suggests tighter and more extensive hydrogen bonding induced by mechanical activation. These interactions likely stabilize the complex and could involve both hydrogen bonding and van der Waals forces, with potential hydrophobic inclusion of aromatic ME constituents [30,41,42].

In the ME/HP-γ-CD systems (Figure 20, Table S9), similar trends were observed.

The 941 cm^−1^ band shifted to 943 cm^−1^ (G) and 945 cm^−1^ (SE), while the 1018 cm^−1^ band moved to 1003 cm^−1^ in both systems. The –OH stretching region also shifted from 3360 cm^−1^ to 3335 cm^−1^ (G) and 3329 cm^−1^ (SE). These consistent redshifts support the presence of hydrogen bonding, although the magnitude suggests these interactions are slightly weaker than in the HP-β-CD system. The large internal cavity of HP-γ-CD may favor hydrophobic interactions with nonpolar ME constituents, especially under SE conditions, which can facilitate better dispersion and alignment of guest molecules within the host matrix [43,44].

In summary, the observed FTIR spectral changes across all ME/CD systems—particularly the shifts in the C–O–C and O–H bands, not visible ME-specific peaks, and the changes in CD-related vibrations—provide evidence of intermolecular interactions, primarily hydrogen bonding. In systems involving hydroxypropylated cyclodextrins, the interactions appear to be more extensive, likely due to their greater flexibility, higher water solubility, and increased number of accessible hydrogen bonding sites. Meanwhile, in native β- and γ-CD systems, the interactions are present but less pronounced, pointing to weaker complexation or partial inclusion. These findings correlate with XRPD results and support the formation of physically stable, supramolecular host–guest structures between ME and the cyclodextrins.

### 2.3. Characterization of Biological Potential of C. islandica Extract/CD Systems

#### 2.3.1. Total Polyphenol Content in *C. islandica* Extract/CD Systems

Polyphenols exert beneficial effects on human health and play an important role in protecting cells from damage caused by oxidative stress [46]. The content and, more importantly, the bioavailability of polyphenols are key criteria for determining the biological potential of tested substances [47]. Systems combining natural compounds (e.g., plant or fungal extracts) with CDs can improve the solubility of low-polarity polyphenols, enhancing their concentration and bioavailability [48,49]. Therefore, the analysis of the TPC in the aqueous fractions of the solubilized CD systems was carried out (Table 4).

The obtained results indicated that complexation of the AE of *C. islandica* with CDs led to the greatest increase in polyphenol content when the SE method was used, particularly in combination with HP-β-CD (13.8 GAE mg/g) and HP-γ-CD (13.6 GAE mg/g). The polyphenol content in the SE system with HP-β-CD increased by 96% compared to the pure extract dissolved in water. When analyzing the ME/CD systems, the highest increase in polyphenol content was observed in the ME complexed with HP-γ-CD using the SE method. The solubility of the pure ME in water was 17.9 GAE mg/g, whereas in the aforementioned system it reached 22.9 GAE mg/g, which represents a 27% increase. These results are presented for *C. islandica* extracts for the first time. However, Jullian et al. (2007) [50] demonstrated that the complexation of quercetin—a polyphenolic flavonoid compound—with three types of CDs (β-CD, HP-β-CD, and SBE-β-CD) significantly improved its solubility, with the greatest enhancement observed for SBE-β-CD and the smallest for β-CD. A different approach regarding the effect of CDs on polyphenols was presented by Vhangani et al. (2022) [51], who reported that the addition of β-CD during the extraction process of *Aspalathus linearis* (green rooibos) increased the stability of polyphenols and enhanced their total content in the obtained extracts, as confirmed by a TPC analysis.

#### 2.3.2. Antioxidant Activity of *C. islandica* Extract/CD Systems

The antioxidant activity of the prepared AE or ME/CD systems and pure extracts dissolved in water was evaluated using the DPPH method. The results indicated that using CDs enhanced certain samples’ activity (Table 5). The most notable improvement was observed for the AE complexed with HP-β-CD, prepared by both SE and G methods. Activity also increased when the AE was complexed with HP-γ-CD. In contrast, complexes of the ME with all four types of CDs did not significantly alter antioxidant activity compared to the pure ME. Moreover, the study results showed that cyclodextrins themselves (β-CD, γ-CD, HP-β-CD, and HP-γ-CD) did not show antioxidant activity, which confirms that the observed effect of the complexes is due solely to the presence of plant extracts.

Lu and coauthors (2009) investigated the antioxidant activity of β-CD–resveratrol (1:1) and HP-β-CD–resveratrol (1:1) complexes using the DPPH method. They observed that increasing the concentration of CDs within the systems resulted in higher antioxidant activity, with HP-β-CD systems showing a stronger effect [52]. These findings are consistent with our results, where systems with HP-β-CD exhibited greater antioxidant activity compared to those with β-CD. Similarly, Iskineyeva et al. (2022) analyzed the antioxidant activity of a resveratrol:β-CD complex (1:2 ratio) using the DPPH assay. They reported a decrease in IC_50_ values following complexation (IC_50_ of resveratrol = 14.3 μg/mL; IC_50_ of resveratrol:β-CD complex = 12.1 μg/mL), confirming that the complexation enhanced the DPPH radical scavenging activity of resveratrol [53]. On the other hand, Chen et al. (2018) described a different phenomenon, a reduction in antioxidant activity upon complexation. In their study, pure curcumin demonstrated a stronger DPPH radical scavenging ability than its inclusion complex with a β-CD–epichlorohydrin polymer. Although increasing the concentration of the complex improved its activity, it remained lower than that of pure curcumin [54].

These findings suggest that both the nature of the active compound and the type of CD used are critical for enhancing solubility and modulating biological activity.

#### 2.3.3. Inhibition of AChE and BChE Enzymes by *C. islandica* Extract/CD Systems

Acetylcholinesterase (AChE) and butyrylcholinesterase (BChE) are enzymes that break down and reduce the level of acetylcholine [55], whose low level is observed during ageing and in individuals with neurodegenerative disorders [56]. For a long time, the substances that inhibit these enzymes have formed a key group of therapeutics used in the treatment of Alzheimer’s disease [57]. Previous studies suggest that compounds present in *C. islandica* extracts, particularly FPCA, possess neuroprotective potential [9]. However, FPCA, like many other secondary lichen metabolites, is poorly soluble in polar solvents [58], and the application of various technological solutions is an important direction of research to increase the bioactivity of lichen extracts. Our results indicated that, compared to aqueous solutions of the pure AE and ME, the aqueous solutions of selected extract/CD systems, particularly those with HP-β-CD and HP-γ-CD, showed enhanced inhibitory activity (Table 6). Notably, greater differences in enzyme inhibition in favor of extract/CD complexes were observed for systems prepared from the ME. To verify whether the observed effects were due solely to the extract or the carrier, the inhibitory activity of the cyclodextrins alone was evaluated. The results showed that native and hydroxypropylated CDs exhibited only marginal AChE and BChE inhibition (generally < 3%). This supports the hypothesis that the increased enzyme inhibition results from improved solubility and availability of active lichen metabolites in the presence of CDs, rather than intrinsic CD activity.

Stasiłowicz-Krzemień et al. (2022) studied the complexation of rosmarinic acid—a neuroprotective depside with poor solubility and low permeability—with various CDs (HP-α-CD, HP-β-CD, and HP-γ-CD) using the SE method. Their experiments demonstrated that, regardless of the type of CD used, the inhibition of both AChE and BChE was significantly increased and plateaued at higher concentrations [29]. These observations are consistent with our findings, where complexes with HP-β-CD and HP-γ-CD enhanced the bioactivity of the examined extracts. However, we noticed the activity loss by systems of extracts with γ-CD. The observed decrease in AChE/BChE inhibition by the SE–γCD complex can probably be attributed to several factors. Firstly, the larger cavity of γ CD (~0.8 nm) [59] allows for the deeper inclusion of bioactive molecules or functional moieties important for enzyme inhibitors contained in extracts [60,61]. Secondly, solvent evaporation fosters highly stable inclusion complexes, potentially hindering the release of active inhibitors [62]. Moreover, enzymes such as AChE and BChE require exposed inhibitor fragments to bind within their deep catalytic gorges; if these fragments are sequestered within γ-CD, inhibition is significantly impaired [63,64,65]. By contrast, tyrosinase inhibition is maintained, as its more accessible active site and different interaction modes render its activity less susceptible to inclusion effects [66]. This underlines the enzyme-specific impact of cyclodextrin complexation. Similarly, other studies demonstrated that β-CD complexation of *Juniperus phoenicea* essential oil improved its aqueous solubility and prolonged its AChE inhibitory activity even in aqueous media [67]. Conversely, Zengin et al. (2023) reported that the complexation of nonpolar *Inula sarana* extracts (n-hexane, ethyl acetate, and dichloromethane) with β-CD resulted in a decrease in biological activity compared to the pure extracts [68].

#### 2.3.4. Inhibition of Tyrosinase by Lichen Extract/CD Systems

Tyrosinase is an enzyme involved in the process of melanogenesis in living organisms [69]. Overactivity of tyrosinase is associated with excessive pigment accumulation in the skin, leading to conditions such as solar lentigo, melasma, and progressive hyperpigmentation [70]. Moreover, elevated tyrosinase expression in the brain can increase the levels of highly reactive melanin precursors, including 3, 4,-dihydroxyphenylalanine (DOPA) and dopaquinone [71]. Thus, tyrosinase inhibition is of interest not only for developing skin-whitening agents but also as a potential strategy to alleviate neurodegeneration associated with Parkinson’s disease [72,73]. Our results showed that, from the perspective of inhibiting tyrosinase activity, the most effective method of complexing AE and ME from *C. islandica* was the SE method. Among the tested CDs, HP-γ-CD demonstrated the greatest enhancement of biological activity. Using the SE method, tyrosinase inhibition increased by 7.43% (a 40% improvement over pure AE) and 8.88% (a 29% improvement over pure ME). In contrast, the use of β-CD and γ-CD, regardless of the method of complexation, did not enhance the inhibitory activity of the extracts (Table 7). The results of studies on the activity of the carriers themselves have proven native and modified CDs exhibited only minimal inhibitory activity (2.8–4.3%) or were inactive (HP-β-CD), indicating that the enhanced activity of the extract/CD systems can be attributed to improved solubility or molecular interactions rather than to any direct enzymatic inhibition by the CDs.

The literature confirms that compounds derived from fungi, including substances present in lichen thalli, can inhibit tyrosinase activity. Experimental studies have demonstrated that both extracts and isolated lichen compounds exert such effects [74]. One of the few reports investigating the effect of CD complexation on tyrosinase activity comes from Stasiłowicz-Krzemień and colleagues (2022) [75]. They studied complexes of the flavonoid naringenin with β-CD and HP-β-CD. Their results showed that the addition of HP-β-CD in a 1:3 molar ratio, prepared either by physical mixing or co-precipitation, increased the inhibitory activity by approximately 19% [75]. Thus, CD complexation effectively enhanced the biological activity of the examined compound.

#### 2.3.5. Dissolution Study of Lichen Extract/CD Systems

**Figure 21:**
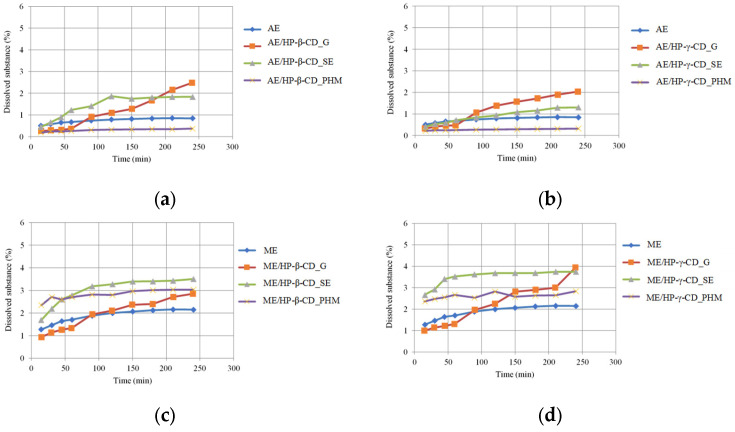
Release profiles (pH 1.2) of fumarprotocetraric acid expressed in % for acetone extract (AE) and AE/HP-β-CD using grinding (G) method, solvent evaporation (SE) method, and with physical mixture (PHM) (**a**); acetone extract (AE) and AE/HP-γ-CD using grinding (G) method, solvent evaporation (SE) method, and with physical mixture (PHM) (**b**); methanol extract (ME) and ME/HP-β-CD using grinding (G) method, solvent evaporation (SE) method, and with physical mixture (PHM) (**c**); methanol extract (ME) and ME/HP-γ-CD using grinding (G) method, solvent evaporation (SE) method, and with physical mixture (PHM) (**d**).

The release rate of FPCA from AE and ME, as well as from extract/HP-β-CD or HP-γ-CD systems selected based on the above-described research, was evaluated by in vitro release tests (Figure 21). The aim of the experiment was to determine whether, under the experimental conditions, the prepared systems could enhance the release of active substances compared to the pure extracts. The dissolution study showed that, regardless of the selected CD, complexes prepared using the G and SE methods for both ME and AE exhibited improved FPCA release profiles compared to pure *C. islandica* extracts (Figure 21). For the AE, better results were achieved with HP-β-CD systems than with HP-γ-CD. Specifically, the system prepared by the G method with HP-β-CD released approximately 2.5% of FPCA (at 240 min), compared to only 0.84% released from the pure AE. On the other hand, the AE/HP-β-CD system prepared using the SE method showed an increased amount of dissolved FPCA (compared to AE) over the longer experimental period (Figure 21a). Conversely, for the ME, the system prepared by the G method with HP-γ-CD exhibited the most favorable release profile: at the final tested time point, 3.94% of FPCA was released, compared to 2.15% from the pure ME. However, the systems prepared with ME/HP-γ-CD by the SE method were characterized by similar FPCA release from 45 min of the study. These results clearly demonstrate the influence of CDs on the release kinetics of FPCA from *C. islandica* extract/CD systems. Although several attempts have been made to combine lichen-derived substances or extracts with carrier systems, most previous studies focused on compounds such as usnic acid. For example, in one analyzed study, the complexation of usnic acid with CDs resulted in an approximately five-fold increase in solubility [76]. However, no previous literature reports have described dissolution rate tests for lichen extracts. Thus, our study should be considered the first to explore this aspect.

#### 2.3.6. Limitations and Assumptions

One limitation of this study is that the calculations for the extract-to-CD ratios were based on the weighed mass of commercial cyclodextrins without correcting for water content. While this could introduce minor variations in the actual amount of active cyclodextrin used, all systems were prepared using the same CD batches, ensuring internal consistency across samples. Furthermore, given the exploratory and comparative nature of this work and the focus on extract-based systems rather than pure compounds, the impact of this simplification is considered minimal. Future studies may benefit from the precise correction of moisture content, particularly in cases where stoichiometric complexation or pharmacokinetic modeling is required.

## 3. Materials and Methods

### 3.1. Plant Material and Reagents

A commercially available raw material was used in this study—the thallus of the lichen *C. islandica*. The methanol extract was prepared using material supplied by Herbal Plant “KAWON–HURT” Nowak sp. j. (Gostyn, Poland), while the acetone extract was prepared using material provided by Herb Packaging Plant “FLOS” Elżbieta and Jan Głąb Sp. j. (Mokrsko, Poland).

Acetone (POCH, Gliwice, Poland); acetonitrile (Sigma-Aldrich, St. Louis, MO, USA); acetylcholinesterase (Sigma-Aldrich, St. Louis, MO, USA); butyrylcholinesterase (Sigma-Aldrich, St. Louis, MO, USA); phosphate buffer pH 6.8 20 x concentrate (STAMAR, Poznań, Poland); magnesium chloride hexahydrate (POCH, Gliwice, Poland); sodium chloride (Sigma-Aldrich, St. Louis, MO, USA); DMSO pure (POCH, Gliwice, Poland); DPPH (2,2-diphenyl-1-picrylhydrazine) (Sigma-Aldrich, St. Louis, MO, USA); ethanol 96% pure (POCH, Gliwice, Poland); acetylthiocholine iodide (Sigma-Aldrich, St. Louis, MO, USA); butyrylthiocholine iodide (Sigma-Aldrich, St. Louis, MO, USA); formic acid 99% pure (Sigma-Aldrich, St. Louis, MO, USA); analytical weight–hydrochloric acid 0.1 mol/L (ALFACHEM Sp. z o.o, Poznań, Poland); methanol pure (POCH, Gliwice, Poland); Folin–Ciocalteu reagent (Merck, Darmstadt, Germany); Tween 80 (Merck, Darmstadt, Germany); Trizma^®^ Base (Sigma-Aldrich, St. Louis, MO, USA); Trizma^®^ Hydrochloride (Sigma-Aldrich, St. Louis, MO, USA); Tyrosinase (Sigma-Aldrich, St. Louis, MO, USA); anhydrous sodium carbonate (POCH, Gliwice, Poland); distilled water. Cyclodextrins: β-cyclodextrin (β-CD; CAS: 7585-39-9, Cat. No. C4767), γ-cyclodextrin (CAS: 17465-86-0; Cat. No. W779547), 2-hydroxypropyl-β-cyclodextrin (CAS: 128446-35-5; Cat. No. 778966), and 2-hydroxypropyl-γ-cyclodextrin (CAS: 128446-34-4; Cat. No. 779229) were purchased from Sigma-Aldrich (St. Louis, MO, USA). Standard substances: galantamine (TRC, Toronto, ON, Canada); ascorbic acid (Sigma-Aldrich, St. Louis, MO, USA); azelaic acid (Sigma-Aldrich, St. Louis, MO, USA); fumarprotocetraric acid (PhytoLab, Vestenbergsgreuth, Germany); gallic acid (Sigma-Aldrich, St. Louis, MO, USA); protocetraric acid (PhytoLab, Vestenbergsgreuth, Germany).

### 3.2. Preparation of Extract

The crushed raw material was weighed (35.5 g) and placed in a ground-glass conical flask. The thalli were extracted separately with 300 mL of acetone or methanol, five times each, using an ultrasonic bath at 40 °C. The obtained filtrates were combined and then evaporated using a vacuum evaporator to dry the residue at a temperature of 40 °C. The extract was stored at 22 °C in a dark place in a tightly closed glass vial.

### 3.3. Phytochemical Characteristic of C. islandica Extracts

#### 3.3.1. High-Performance Liquid Chromatography (HPLC) Analyses

The quantification of *C. islandica* extracts was effectuated using the High-Performance Liquid Chromatography (HPLC) method described by Studzińska-Sroka et al. (2021) with some modifications [10]. The HPLC system (Thermo Scientific Dionex UltiMate 3000 UHPLC, Sunnyvale, CA, USA) was used to perform the quantitative analysis. Separations were performed on a ReproShell ODS-3, 5 µm, 100 × 2 mm (Dr. Maisch). The mobile phase (flow rate 0.5 mL/min) consisted of a mixture of solution A (0.5% formic acid) and solution B (acetonitrile). The gradient elution started from 5% of acetonitrile to 100% during 10 min, and then the isocratic elution with 100% acetonitrile was performed for 2 min. From 12 min to 17 min, the concentration of acetonitrile decreased to 5% and the column was re-equilibrated to starting conditions for 3 min. The wavelength of the diode array detector (DAD) was 254 nm. The column was at 40 °C. The results were presented as the average of three independent measurements ± standard deviation (SD). The method was validated for FPCA and for PCA. The HPLC method was validated according to ICH guidelines, and validation parameters are collected in Table S10 (Supplementary Materials).

#### 3.3.2. Total Polyphenol Content (TPC)

A TPC analysis was carried out according to Studzińska-Sroka et al. (2021) [77]. In a 96-well plate, 25.0 µL of the extract samples (4 mg/mL AE, 5 mg/mL ME) dissolved in DMSO or 25.0 µL of standard (gallic acid at concentrations 5–160 µg/mL), 200 µL of distilled water, 15 µL of Folin–Ciocalteu reagent, and 60 µL of Na_2_CO_3_ (20% solution in distilled water) were mixed. The 25.0 µL of water was added instead of the sample to prepare the blank. The plate was agitated at 600 rpm for 10 min and then incubated for 20 min. The incubation was performed at room temperature in the dark. The absorbance was read using a plate reader at 760 nm wavelength (spectrophotometer UV/VIS, Lambda 35, Elmer–Perkin). The experiment was carried out in duplicate. The results are the average of *n* = 6 measurements for the samples and of *n* = 5 measurements for the standard. The TPC was expressed as mg gallic acid equivalent (GAE) per g of a dry extract ± SD.

#### 3.3.3. Fourier Transform Infrared Spectroscopy with Attenuated Total Reflectance (FTIR-ATR)

The ATR-FTIR spectra of FPCA, AE, and ME solid samples were collected on an IRTracer-100 spectrophotometer (measurement parameters: resolution: 4 cm^−1^, number of scans: 100, and range: 400–1800 cm^−1^). Using LabSolutions IR software (version 1.86 SP2, Shimadzu, Kyoto, Japan), the second derivative of each spectrum was determined. Calculations were carried out by the software using the Savitzky–Golay numerical technique. Eleven points were the smoothing parameter. To analyze the collected data, Origin 2021b (OriginLab Corporation, Northampton, MA, USA) was utilized.

The ATR-FTIR spectra of AE/ME extract, CD (β-CD, γ-CD, HP-β-CD, and HP-γ-CD), AE/CD systems, ME/CD systems, PHMs of AE/CD, and PHMs of ME/CD solid samples were collected on an IRTracer-100 spectrophotometer (measurement parameters: resolution: 4 cm^−1^, number of scans: 100, apodization: Happ–Genzel, and range: 400–4000 cm^−1^). With LabSolution IR software (version 1.86 SP2, Shimadzu, Kyoto, Japan), all spectra were collected and then processed, including baseline correction and normalization. Origin 2021b (OriginLab Corporation, Northampton, MA, USA) was used to analyze the acquired data.

#### 3.3.4. GC-MS Analysis of the *C. islandica* Extracts

Chromatographic studies were performed on a GC-MS chromatograph (SCION TQ, BRUKER, Billerica, MA, USA). A total of 10.0 µL of the sample was dissolved in 2.0 mL of CH_2_Cl_2_ (Sigma-Aldrich), and 1.0 µL of the solution was injected onto the column. The chromatograph was equipped with a VF-5ms Crawford Scientific silica column. The electron energy was 70 eV, and the ion source was at 200 °C. Helium was used as the carrier gas at a flow rate of 1.0 mL/min. Temperature program: enable coolant at 50.0 °C, coolant timeout 20.00 min, stabilization time 0.50 min.; temperature 60.0 °C, hold 3.00 min, total 3.00 min; temperature 280.0 °C, rate 10.0 °C/min, hold 35.00 min, total 60.00 min. The identification of compounds was based on a comparison of their retention time as well as mass spectra with NIST standards.

### 3.4. System Preparation

The systems of AE and ME were prepared using both unmodified (β-CD and γ-CD) and hydroxypropylated cyclodextrins (HP-β-CD and HP-γ-CD) using two methods: the SE and G methods [30,78]. Unmodified CDs (with water content 11% for β-CD and 8% for γ-CD) and hydroxypropylated CDs (with declared degrees of substitution: DS = 0.9 for HP-β-CD and 0.6 for HP-γ-CD and water content: 3.9% for HP-β-CD and 1.9% for HP-γ-CD) were used as received from the supplier. Calculations were based on weighed masses without correcting for water content; however, all systems were prepared using the same CD batches to ensure consistency.

The extract-to-CD mass ratio of 1:1.5 was selected based on the experimentally determined content of the active compound (FPCA) in the complex, which reached approximately 26%. Due to the complex nature of the extract and the diversity of compound molecular weights, the mass ratio was used instead of a molar ratio to ensure reproducibility and process feasibility.

For the SE method, an accurately weighed amount of CD was dissolved in a specific volume of water, obtaining an aqueous 1% β-CD solution and 10% γ-CD solution, HP-β-CD, and HP-γ-CD. The AE (100 mg) was dissolved in 100 mL of acetone (1 mg/mL solution), and the ME (200 mg) was dissolved in 100 mL of methanol (2 mg/mL solution). The extracts were added dropwise to the dissolved CD. The mass ratio of extracts and selected CD was 1:1.5. The system was evaporated to dryness under reduced pressure. The process was performed at 60 °C.

For the G method, an accurately weighed amount of CD and an accurately weighed amount of dry extracts in a mass ratio of 1:1 were transferred to an agate mortar and ground with a pestle for two hours. After this time, another weighed portion of CD was added so that the mass ratio of the extract to CD was 1:1.5. The contents of the mortar were ground for another 1 h.

To prepare PHMs, CDs, and *C. islandica*, AE and ME were weighed, and each extract was combined with each CD so that the mass ratio of extracts and selected CDs was 1:1.5. All components were then mechanically mixed.

### 3.5. Identification of C. islandica Extract/CD Systems

#### 3.5.1. X-Ray Powder Diffraction Analysis (XRPD)

XRD equipment (Panalytical Empyrean, Almelo, The Netherlands) with a copper anode (CuKα—1.54 Å). The measurements were conducted in Bragg–Brentano reflection mode configuration with parameters set to 45 kV and 40 mA. The measurement range was defined from 5° to 40°, with a step size of 0.05° and a measurement time of 45 s per step.

Based on the XRPD data, we determine the Crystallinity Index (CI) of the analyzed samples; the sum of the area under the crystalline peaks was compared to the total area under the XRPD pattern, which includes both crystalline and amorphous contributions. As stated in previous studies [79], “at various degrees of crystallinity, the demarcation of the crystalline and amorphous intensities are difficult and subjected to individual judgement”. To minimize such errors, particularly for samples with a high degree of crystallinity (such as β-CD, γ-CD, and AE, ME), only PXRD peaks with intensities greater than 20% of the most intense peak were considered in the calculation. Peak fitting and area integration were performed using Origin 2021b software. The Crystallinity Index (CI) in the blends was calculated according to Equation (1) [80]:

CI% = (CA/TA) × 100%(1)

where CI = Crystallinity Index; CA = area under crystallinity peak; and TA = total area.

#### 3.5.2. Fourier Transform Infrared Spectroscopy with Attenuated Total Reflectance (FTIR-ATR)

The FTIR-ATR was used to characterize the *C. islandica* extract/CD systems. The methodology was presented in Section 3.3.3.

### 3.6. Biological Activity of C. islandica Extract/CD Systems

#### 3.6.1. Total Phenolic Content (TPC) of Extract/CD Systems

The analysis was carried out according to Studzińska-Sroka et al. (2021) [77]. The general methodology was presented in Section 3.3.2, with the exception that extracts and extract/CD systems were dissolved in water at a concentration of 4 mg of extract/mL for AE and 5 mg/mL for ME and systems, and the supernatant above the sediment was used for the assay. The results are the average of n = 6 measurements.

#### 3.6.2. Antioxidant Activity

According to Studzińska-Sroka et al. (2021) [77], 25 µL of the test sample and 175 µL of DPPH radical methanolic solution were placed on a 96-well plate. Extracts and extract/CD systems were dissolved in water at a concentration of 20 mg of extract/mL, and the supernatant above the sediment was used for the assay if the sample was not completely dissolved. At the same time, a blank (water instead of a sample and methanol instead of a DPPH solution), a control (water instead of a sample), and a blank for tested samples (methanol instead of a DPPH solution) for the examined samples were carried out. Then, the plate was incubated for 30 min with shaking (25 °C, 600 rpm, in the dark). The activity of individual cyclodextrins was also tested as an internal control to assess any potential CD-related radical scavenging activity or interference. The values were presented alongside the extract–CD complexes for comparison. The experiment was carried out in duplicate. The results are the average of n = 5 measurements. The absorbance was measured at a 517 nm wavelength. The ability to reduce the DPPH radical was calculated using the following formula:

DPPH^•^ scavenging ability (%) = [(A_0_ − A_1_)/A_0_] × 100%

where A_0_ is the absorbance of the control and A_1_ is the absorbance of the tested sample.

#### 3.6.3. Enzymatic Activity

##### Effect on Acetylcholinesterase (AChE) and Butyrylcholinesterase (BChE) Activity

Cholinesterase activities were assessed using a method described in the literature with modification [81]. The determination was performed on a 96-well plate by adding 5 µL of the test sample, 60 µL of TRIS-HCl buffer (pH 8), and 30 µL of the AChE or BChE solution (0.2 U/mL). Extracts and extract/CD systems were dissolved in water at a concentration of 40 mg of extract/mL, and the supernatant above the sediment was used for the assay if the sample was not completely dissolved. The prepared plate was incubated for 5 min in the dark and at room temperature with shaking (600 rpm). After the first incubation stage, 30 µL of acetylthiocholine iodide (ACTI) or butyrylthiocholine iodide (BTCI) solution (1.5 mM) and 125 µL of DTNB were added to the wells and incubated for the next 30 or 20 min, respectively (in dark and at room temperature with shaking 600 rpm). At the same time, a blank (with water instead of the sample and with a buffer instead of the enzyme), a control (with water instead of the sample), and a blank for test samples (with buffer without the enzyme) were prepared. The activity of individual cyclodextrins on each enzyme was also tested as an internal control to assess potential CD-related interference. The values were presented alongside the extract–CD complexes for comparison. The experiment was carried out in duplicate. The results are the average of n = 4 measurements. The absorbance was measured at a 405 nm wavelength. The ability to the inhibition of the enzyme was calculated using the following formula:

AChE or BChE inhibition (%) = [(A_0_ − A_1_)/A_0_] × 100%

where A_0_ is the absorbance of the control and A_1_ is the absorbance of the tested sample.

##### Effect on Tyrosinase Activity

The analysis was performed in accordance with Studzińska-Sroka et al. (2024) [82] on a 96-well plate by applying the following: 25 µL of the test, 75 µL of phosphate buffer (pH 6.8), and 50 µL of tyrosinase solution (192 U/mL). Extracts and extract/CD systems were dissolved in water at a concentration of 8 mg of extract/mL, and the supernatant above the sediment was used for the assay if the sample was not completely dissolved. The plate was incubated for 10 min at room temperature and in the dark (with shaking, 600 rpm). After the first stage of incubation, 50 µL of L-DOPA solution (2 mM) was added to the wells of the test sample. The plate was incubated for the next 20 min, also at room temperature, with shaking (600 rpm) and in dark conditions. At the same time, a blank (with water instead of the sample and with buffer instead of the enzyme), a control (with water instead of the sample), and a blank for test samples (with a buffer without the L-DOPA) were prepared. The activity of individual cyclodextrins on the enzyme was also tested as an internal control to assess potential CD-related interference. The values were presented alongside the extract–CD complexes for comparison. The experiment was carried out in duplicate. The results are the average of n = 5 measurements. The absorbance was measured at 475 nm wavelength. The ability to the inhibition of the enzyme was calculated using the following formula:

Tyrosinase inhibition (%) = [(A_0_ − A_1_)/A_0_] × 100%

where A_0_ is the absorbance of the control and A_1_ is the absorbance of the tested sample.

### 3.7. Dissolution Study

Dissolution studies of AE and ME and CD systems (HP-β-CD and HP-γ-CD) were carried out using an Agilent 708-DS (Santa Clara, CA, USA) dissolution apparatus. Either 50 mg of the pure extract or an amount of the extract/CD system equivalent to 50 mg of extract was weighed and placed into a gelatin capsule. The capsules were placed in 250 mL of acceptor solution containing 0.1 mol/L HCl with Tween 80 (0.1%). The analysis was performed at 37 ± 0.5 °C with a stirring speed of 50 rpm. Samples (2 mL) were collected every 15 min for the first hour, then every 30 min—the study lasted a total of 4 h. The collected volume was replaced with 2 mL of media. The samples were filtered using a 0.45 µm nylon membrane filter (Sigma-Aldrich, St. Louis, MO, USA). The concentrations of FPCA in the filtered acceptor solutions were determined using the HPLC method described above, with respect to the three injections of 30 µL that were performed for each sample. The results were presented as % of released FPCA.

### 3.8. Statistical Analysis

The means ± SD used to express the collected data were calculated using Microsoft Excel (Microsoft Office LTSC 2021 Professional Plus, Microsoft Corp., Redmond, WA, USA). A one-way analysis of variance (ANOVA) was used for statistical analysis, and Statistica software (version 13.3, StatSoft, Krakow, Poland) was used to calculate statistical differences (using Duncan’s post hoc tests) with a significance threshold of *p* < 0.05.

## 4. Conclusions

In conclusion, the obtained results demonstrated that CD carriers can interact with components of *C. islandica* extracts and enhance the biological activity of FPCA-rich extracts (AE and ME). The most promising effects—stronger interactions and increased biological activity—were observed in systems combining *C. islandica* extracts with HP-β-CD and HP-γ-CD. Notably, ME-based systems exhibited higher antioxidant and enzyme inhibitory activity (AChE, BChE, and tyrosinase) than the AE-based counterparts despite containing less FPCA. This study also revealed that combinations of *C. islandica* extracts with HP-β-CD and HP-γ-CD exhibited improved FPCA release profiles. Although cyclodextrins alone exhibited negligible or no inhibitory activity toward AChE, BChE, and tyrosinase and showed no antioxidant activity, their inclusion complexes with *C. islandica* extracts significantly enhanced enzymatic inhibition, indicating that the observed bioactivity results from improved solubility and molecular interactions rather than intrinsic effects of the carriers. Additionally, it was observed that the ME, despite containing a lower amount of FPCA, released more depsidones compared to the more lipophilic AE.

To the best of our knowledge, this is the first study to report the preparation and characterization of CD-based systems with *C. islandica* extracts, and the findings support further research into the use of such delivery systems to enhance the biological potential of lichen-derived compounds in medicine.

## Figures and Tables

**Table 1:** Compounds identified in the extracts of *C. islandica* using GC-MS.

Extract	Rt (min.)	Compounds	% of Total	MW	Formula
Acetone extract	18.468	Tricyclo [5.4.3.0(1,8)]tetradecan-6-one, 4-ethenyl-3-hydroxy-2,4,7,14-tetramethyl	1.019	304.46	C_20_H_32_O_2_
	19.512	Hexadecanoic acid, 1-(hydroxymethyl)-1,2-ethanediyl ester	1.876	568.91	C_35_H_68_O_5_
	20.837	Cholestan-3-one, cyclic 1,2-ethanediyl aetal, (5ß)-	5.149	430.70	C_29_H_50_O_2_
	21.142	12-Methyl-E,E-2,13-octadecadien-1-ol	2.354	280.49	C_19_H_36_O
	21.242	Oleic Acid	6.710	282.46	C_18_H_34_O_2_
	21.496	7,8-Epoxylanostan-11-ol, 3-acetoxy-	4.006	502.76	C_32_H_54_O_4_
	21.723	Oxiraneoctanoic acid, 3-octyl-, cis-	7.108	298.46	C_18_H_34_O_3_
	21.979	8-Hexadecenal, 14-methyl-, (Z)-	64.164	252.43	C_17_H_32_O
	23.735	15,17,19,21-Hexatriacontatetrayne	7.615	490.84	C_36_H_58_
Total			100.00		
Methanol extract	18.117	14-Octadecenal	1.21	266.46	C_18_H_34_O
	19.438	n-Hexadecanoic acid	5.99	256.42	C_16_H_32_O_2_
	20.688	7,10-Octadecadienoic acid, methyl ester	6.23	294.47	C_19_H_34_O_2_
	21.070	Linoleic acid	32.88	280.44	C_18_H_32_O_2_
	21.495	Methyl kauran-18-oate	4.76	318.49	C_21_H_34_O_2_
	21.973	2-Methyl-Z,Z-3,13-octadecadienol	24.29	280.49	C_19_H_36_O
	22.221	Oxiraneoctanoic acid, 3-octyl-, cis-	3.92	298,461	C_18_H_34_O_3_
	23.252	Cholesta-5,7,9(11)-trien-3-ol acetate	5.34	424.66	C_29_H_44_O_2_
	23.747	Olean-12-ene-3,15,16,21,22,28-hexol, (3ß,15a,16a,21ß,22a)-	4.07	506.71	C_30_H_50_O_6_
	24.902	Pregna-4,6-diene-3,20-dione, 17-(acetyloxy)-6,16-dimethyl-, (16a)-	11.31	398.53	C_25_H_34_O_4_
Total			100.00		

**Table 2:** Crystallinity Index (CI) values of AE and CD calculated from the XRPD data.

		CI (%)		
		**raw**	**SE**	**G**
**AE/β-CD**	**β-CD**	55.52	33.46	47.17
	**AE**	56.96	32.45	19.20
**AE/γ-CD**	**γ-CD**	84.16	27.33	65.02
	**AE**	56.96	27.13	15.63
**AE/HP-β-CD**	**AE**	56.96	7.74	19.94
**AE/HP-γ-CD**	**AE**	56.96	22.83	6.14

**Table 3:** Crystallinity Index (CI) values of ME and CD calculated from the XRPD data.

		CI (%)		
		**raw**	**SE**	**G**
**ME/β-CD**	**β-CD**	55.52	34.74	2.79
	**ME**	72.32	19.65	63.43
**ME/γ-CD**	**γ-CD**	84.16	36.13	72.05
	**ME**	72.32	25.36	5.11
**ME/HP-β-CD**	**ME**	72.32	1.88	6.78
**ME/HP-γ-CD**	**ME**	72.32	8.61	4.34

**Table 4:** Total Polyphenol Content in *Cetraria islandica* acetone and methanol extracts and in extract/cyclodextrins systems when H_2_O is used as a solvent.

Tested Sample		Total Polyphenol Content (GAE mg/g)			
		UE	G	SE	PHM
**Acetone** **extract**	**AE**	7.0 ± 0.1 ^h^	-	-	-
	**β-CD**	-	7.7 ± 0.1 ^f,g^	10.7 ± 0.1 ^c^	7.5 ± 0.1 ^g^
	**γ-CD**	-	7.6 ± 0.2 ^f,g^	7.7 ± 0.3 ^f,g^	7.9 ± 0.3 ^f^
	**HP-β-CD**	-	9.6 ± 0.3 ^d^	13.8 ± 0.4 ^a^	7.9 ± 0.3 ^f^
	**HP-γ-CD**	-	12.1 ± 0.2 ^b^	13.6 ± 0.2 ^a^	9.0 ± 0.2 ^e^
**Methanol** **extract**	**ME**	17.9 ± 0.5 ^e^	-	-	-
	**β-CD**	-	18.7 ± 0.6 ^d^	16.8 ± 0.6 ^f^	16.4 ± 0.3 ^f^
	**γ-CD**	-	18.6 ± 0.4 ^d,e^	16.7 ± 0.9 ^f^	18.1 ± 1.0 ^d,e^
	**HP-β-CD**	-	21.2 ± 0.4 ^b^	20.2 ± 0.6 ^c^	17.9 ± 0.2 ^e^
	**HP-γ-CD**	-	21.3 ± 0.5 ^b^	22.9 ± 0.2 ^a^	21.6 ± 0.2 ^b^

AE—acetone extract; ME—methanol extract; UE—ultrasonic extraction; G—grinding method; SE—solvent evaporation method; PHM—physical mixture. Mean values within a column with the same letter are not significantly different at *p* < 0.05 using Duncan’s test. The first letter of the alphabet for the highest inhibitory activity.

**Table 5:** The antioxidant activity (DPPH method) of *Cetraria islandica* acetone and methanol extracts and extract/cyclodextrin systems when H_2_O is used as solvent.

Tested Sample		Antioxidant Activity (%)				
		UE	G	SE	PHM	CD
**Acetone** **extract**	**AE**	66.0 ± 1.3 ^c^	-	-	-	-
	**β-CD**	-	68.9 ± 2.6 ^c^	62.8 ± 0.9 ^d^	68.9 ± 1.9 ^c^	na
	**γ-CD**	-	56.8 ± 3.3 ^e^	66.1 ± 5.5 ^c^	62.0 ± 1.9 ^d^	na
	**HP-β-CD**	-	73.9 ± 2.5 ^b^	78.9 ± 1.4 ^a^	62.6 ± 1.4 ^d^	na
	**HP-γ-CD**	-	67.5 ± 1.2 ^c^	73.7 ± 0.7 ^b^	52.1 ± 0.9 ^f^	na
**Methanol** **extract**	**ME**	93.2 ± 0.2 ^b,c^	-	-	-	-
	**β-CD**	-	93.1 ± 2.0 ^b,c^	88.1 ± 0.4 ^d^	92.5 ± 0.2 ^c^	na
	**γ-CD**	-	82.0 ± 2.1 ^f^	87.6 ± 1.3 ^d^	85.4 ± 0.8 ^e^	na
	**HP-β-CD**	-	93.6 ± 0.1 ^a,b,c^	92.8 ± 0.3 ^b,c^	94.5 ± 0.2 ^a^	na
	**HP-γ-CD**	-	93.7 ± 0.2 ^a,b^	93.3 ± 0.2 ^b,c^	93.5 ± 0.3 ^a,b,c^	na

AE—acetone extract; ME—methanol extract; UE—ultrasonic extraction; G—grinding method; SE—solvent evaporation method; PHM—physical mixture; CD—cyclodextrin; na—not active. Mean values within a column with the same letter are not significantly different at *p* < 0.05 using Duncan’s test. The first letter of the alphabet for the highest inhibitory activity.

**Table 6:** The AChE and BChE inhibitory activity of *Cetraria islandica* acetone and methanol extracts and extract/cyclodextrin systems when H_2_O is used as solvent.

Tested Sample		AChE Inhibition (%)					BChE Inhibition (%)				
		UE	G	SE	PHM	CD	UE	G	SE	PHM	CD
**Acetone** **extract**	**AE**	3.1 ± 1.2 ^d,e,f^	-	-	-	-	9.8 ± 1.9 ^b,c^	-	-	-	-
	**β-CD**	-	4.0 ± 0.3 ^d,e^	4.6 ± 1.6 ^d^	8.9 ± 1.3 ^b,c^	2.6 ± 2.7 ^e,f,g^	-	5.8 ± 1.0 ^d^	5.6 ± 0.3 ^d^	6.2 ± 1.7 ^d^	1.4 ± 0.7 ^h^
	**γ-CD**	-	3.6 ± 1.1 ^d,e^	na	1.3 ± 0.2 ^f,g,h^	0.24 ± 0.7 ^g^	-	3.5 ± 1.1 ^g^	na	3.5 ± 0.7 ^g^	0.5 ± 1.1 ^h^
	**HP-β-CD**	-	10.9 ± 0.3 ^a^	3.2 ± 1.5 ^d,e^	9.9 ± 0.6 ^a,b^	na	-	12.1 ± 1.7 ^a^	10.7 ± 1.0 ^a,b^	8.9 ± 0.7 ^c^	na
	**HP-γ-CD**	-	4.7 ± 1.4 ^d^	7.9 ± 0.6 ^c^	8.1 ± 1.3 ^b,c^	0.80 ± 0.5 ^f,g^	-	11.1 ± 1.1 ^a,b^	11.8 ± 0.7 ^a^	7.1 ± 0.9 ^d^	1.73 ± 1.0 ^h^
**Methanol** **extract**	**ME**	3.9 ± 1.0 ^g^	-	-	-	-	7.2 ± 0.7 ^f^	-	-	-	-
	**β-CD**	-	2.01 ± 0.7 ^h,i^	5.6 ± 0.4 ^f^	11.0 ± 0.4 ^c,d^	2.6 ± 2.7 ^g,h^	-	9.0 ± 1.3 ^d,e^	7.6 ± 1.4 ^e,f^	9.8 ± 0.6 ^d^	1.4 ± 0.7 ^h^
	**γ-CD**	-	3.6 ± 0.4 ^g^	1.6 ± 0.7 ^h,i,j^	10.0 ± 0.3 ^d^	0.24 ± 0.7 ^j^	-	3.9 ± 0.4 ^g^	5.1 ± 1.7 ^g^	7.8 ± 0.5 ^e,f^	0.5 ± 1.1 ^h^
	**HP-β-CD**	-	10.8 ± 0.5 ^c,d^	7.7 ± 0.5 ^e^	11.5 ± 0.7 ^c^	na	-	13.3 ± 2.0 ^b,c^	14.2 ± 1.5 ^b^	14.3 ± 1.3 ^b^	na
	**HP-γ-CD**	-	11.9 ± 0.8 ^c^	23.9 ± 0.7 ^a^	20.9 ± 0.9 ^b^	0.80 ± 0.5 ^i,j^	-	12.6 ± 1.2 ^c^	16.7 ± 1.2 ^a^	14.3 ± 0.7 ^b^	1.73 ± 1.0 ^h^

AE—acetone extract; ME—methanol extract; UE—ultrasonic extraction; G—grinding method; SE—solvent evaporation method; PHM—physical mixture; CD—cyclodextrin; na—not active. Mean values within a column with the same letter are not significantly different at *p* < 0.05 using Duncan’s test. The first letter of the alphabet for the highest inhibitory activity.

**Table 7:** The tyrosinase inhibitory activity of *Cetraria islandica* acetone and methanol extracts and extract/cyclodextrin systems when H_2_O is used as solvent.

Tested Sample		Tyrosinase Inhibition (%)				
		UE	G	SE	PHM	CD
**Acetone** **extract**	**AE**	18.8 ± 2.6 ^c^	-	-	-	-
	**β-CD**	-	13.9 ± 2.0 ^d^	20.6 ± 2.0 ^b,c^	10.2 ± 1.1 ^e^	2.8 ± 0.5 ^f^
	**γ-CD**	-	13.8 ± 1.8 ^d^	22.3 ± 1.6 ^b^	14.9 ± 1.4 ^d^	4.3 ± 1.4 ^f^
	**HP-β-CD**	-	20.9 ± 1.5 ^b,c^	25.9 ± 1.6 ^a^	15.9 ± 4.1 ^d^	na
	**HP-γ-CD**	-	22.5 ± 0.3 ^b^	26.2 ± 0.7 ^a^	21.5 ± 0.4 ^b^	2.8 ± 0.7 ^f^
**Methanol** **extract**	**ME**	25.3 ± 0.8 ^c,d^	-	-	-	-
	**β-CD**	-	19.4 ± 1.5 ^h^	25.4 ± 0.8 ^c,d^	23.4 ± 0.7 ^e^	2.8 ± 0.5 ^j^
	**γ-CD**	-	21.0 ± 0.5 ^f,g^	24.9 ± 0.4 ^c,d^	19.9 ± 0.8 ^g,h^	4.3 ± 1.4 ^i^
	**HP-β-CD**	-	24.8 ± 0.7 ^d^	32.62 ± 2.2 ^b^	21.9 ± 0.7 ^f^	na
	**HP-γ-CD**	-	26.2 ± 1.0 ^c^	34.2 ± 0.5 ^a^	21.4 ± 0.6 ^f^	2.8 ± 0.7 ^j^

AE—acetone extract; ME—methanol extract; UE—ultrasonic extraction; G—grinding method; SE—solvent evaporation method; PHM—physical mixture; CD—cyclodextrin; na—not active. Mean values within a column with the same letter are not significantly different at *p* < 0.05 using Duncan’s test. The first letter of the alphabet for the highest inhibitory activity.

## Data Availability

All data supporting the reported results can be found within the manuscript and Supplementary Materials.

## Supplementary Materials

The following supporting information can be downloaded at https://www.mdpi.com/article/10.3390/molecules30153182/s1, Figure S1: The results of FTIR analysis (3800–2600 cm^−1^) of acetone extract (AE) (black), β-cyclodextrin (β-CD) (red), AE/β-CD 1:1.5 physical mixture (PHM) (blue), AE/β-CD 1:1.5 system by grinding (G) (yellow), and AE/β-CD 1:1.5 system by solvent evaporation (SE) (green) (a); The results of FTIR analysis (3800–2600 cm^−1^) of acetone extract (AE) (black), γ-cyclodextrin (γ-CD) (red), AE/γ-CD 1:1.5 physical mixture (PHM) (blue), AE/γ-CD 1:1.5 system by grinding (G) (yellow), and AE/γ-CD 1:1.5 system by solvent evaporation (SE) (green) (b); The results of FTIR analysis (3800–2600 cm^−1^) of acetone extract (AE) (black), 2-hydroxypropyl-β-cyclodextrin (HP-β-CD) (red), AE/HP-β-CD 1:1.5 physical mixture (PHM) (blue), AE/HP-β-CD 1:1.5 system by grinding (G) (yellow), and AE/HP-β-CD 1:1.5 system by solvent evaporation (SE) (green) (c); The results of FTIR analysis (3800–2600 cm^−1^) of acetone extract (AE) (black), hydroxypropyl-γ-cyclodextrin (HP-γ-CD) (red), AE/HP-γ-CD 1:1.5 physical mixture (PHM) (blue), AE/HP-γ-CD 1:1.5 system by grinding (G) (yellow), and AE/HP-γ-CD 1:1.5 system by solvent evaporation (SE) (green) (d); Table S1: Selected characteristic bands in second derivative FTIR spectra of fumarprotocetraric acid, AE, and ME; Table S2: Selected characteristic bands (in cm^−1^) of acetone extract (AE), β-cyclodextrin (β-CD), and physical mixture (PHM) of AE and β-CD (PHM) for which changes were observed in AE/β-CD system prepared by grinding (G) and AE/β-CD system prepared by solvent evaporation (SE); Table S3: Selected characteristic bands (in cm^−1^) of acetone extract (AE), γ-cyclodextrin (γ-CD), and physical mixture (PHM) of AE and γ-CD for which changes were observed in AE/γ-CD system prepared by grinding (G) and AE/γ-CD system prepared by solvent evaporation (SE); Table S4: Selected characteristic bands (in cm^−1^) of acetone extract (AE), HP-β-CD-cyclodextrin (HP-β-CD), and physical mixture (PHM) of AE and HP-β-CD for which changes were observed in AE/HP-β-CD system prepared by grinding (G) and AE/HP-β-CD system prepared by solvent evaporation (SE); Table S5: Selected characteristic bands (in cm^−1^) of acetone extract (AE), HP-γ-CD-cyclodextrin (HP-γ-CD), and physical mixture (PHM) of AE and HP-γ-CD for which changes were observed in AE/HP-γ-CD system prepared by grinding (G) and AE/HP-γ-CD system prepared by solvent evaporation (SE); Table S6: Selected characteristic bands (in cm^−1^) of methanol extract (ME), β-cyclodextrin (β-CD), and physical mixture (PHM) of ME and β-CD for which changes were observed in ME/β-CD system prepared by grinding (G) and ME/β-CD system prepared by solvent evaporation (SE); Table S7: Selected characteristic bands (in cm^−1^) of methanol extract (ME), γ-CD-cyclodextrin (γ-CD) and physical mixture (PHM) of ME and γ-CD for which changes were observed in ME/γ-CD system prepared by grinding (G) and ME/γ-CD system prepared by solvent evaporation (SE); Table S8: Selected characteristic bands (in cm^−1^) of methanol extract (ME), HP-β-CD-cyclodextrin (HP-β-CD), and physical mixture (PHM) of ME and HP-β-CD for which changes were observed in ME/HP-β-CD system prepared by grinding (G) and ME/HP-β-CD system prepared by solvent evaporation (SE); Table S9: Selected characteristic bands (in cm^−1^) of methanol extract (ME), HP-γ-CD-cyclodextrin (HP-γ-CD), and physical mixture of ME and HP-γ-CD (PHM) for which changes were observed in ME/HP-γ-CD system prepared by grinding (G) and ME/HP-γ-CD system prepared by solvent evaporation (SE); Table S10: HPLC method validation parameters.
