# Supplementary material for: Cyclodextrin-Based Systems of Cetraria islandica Extracts: A Novel Approach to Improve Solubility and Biological Activity of Lichen-Derived Natural Products
Source: Molecules. 2025 Jul 29;30(15):3182. doi: 10.3390/molecules30153182 (PMC12348940; doi:10.3390/molecules30153182)
Supplement: Supplementary file 1 [file molecules-30-03182-s001.zip › molecules-3660521-supplementary.pdf]

molecules

Supplementary Materials: Cyclodextrin-Based Systems of *Cetraria islandica* Extracts: A Novel Approach to Improve Solubility and Biological Activity of Lichen-Derived Natural Products

Elżbieta Studzińska-Sroka [1,*], Karolina Cichoracka [1], Natalia Rosiak [1], Andrzej Miklaszewski [2], Marcin Szymański [3] and Judyta Cielecka-Piontek [1]

[1] Department of Pharmacognosy and Biomaterials, Poznan University of Medical Sciences, Rokietnicka 3 Str, 60-806 Poznan, Poland; karolina.pesta99@gmail.com (K.C.); nrosiak@ump.edu.pl (N.R.); jpiontek@ump.edu.pl (J.C.-P.)
[2] Faculty of Materials Engineering and Technical Physics, Institute of Materials Science and Engineering, Poznan University of Technology, 60-965 Poznan, Poland; andrzej.miklaszewski@put.poznan.pl
[3] Center for Advanced Technologies, Adam Mickiewicz University in Poznań, Uniwersytetu Poznańskiego 10 Str, 61-614 Poznan, Poland; marcin.szymanski@amu.edu.pl
* Correspondence: elastudzinska@ump.edu.pl

Table S1. Selected characteristic bands in second derivative FTIR spectra of fumarprotocetraric acid, acetone extract, and methanol extract

fumarprotocetraric acid [cm^{-1}]	acetone extract [cm^{-1}]	methanol extract [cm^{-1}]
457	457	-
476	476	-
500	498	-
530	530	528
565	565	-
598	598	598
679	679	-
702	700	-
760	760	762
783	783	783
800	800	802
843	841	-
870	868	868
1047	1047	-
1067	1067	-
1088	1088	-
1128	1128	-
1152	1153	1153
1206	1206	1204
1231	1231	1231
1254	1254	1254
1294	1294	1294

1317	1315	1315
1358	1358	1358
1379	1379	-
1395	1395	-
1414	1414	1416
1574	-	1574
1611	1611	1611

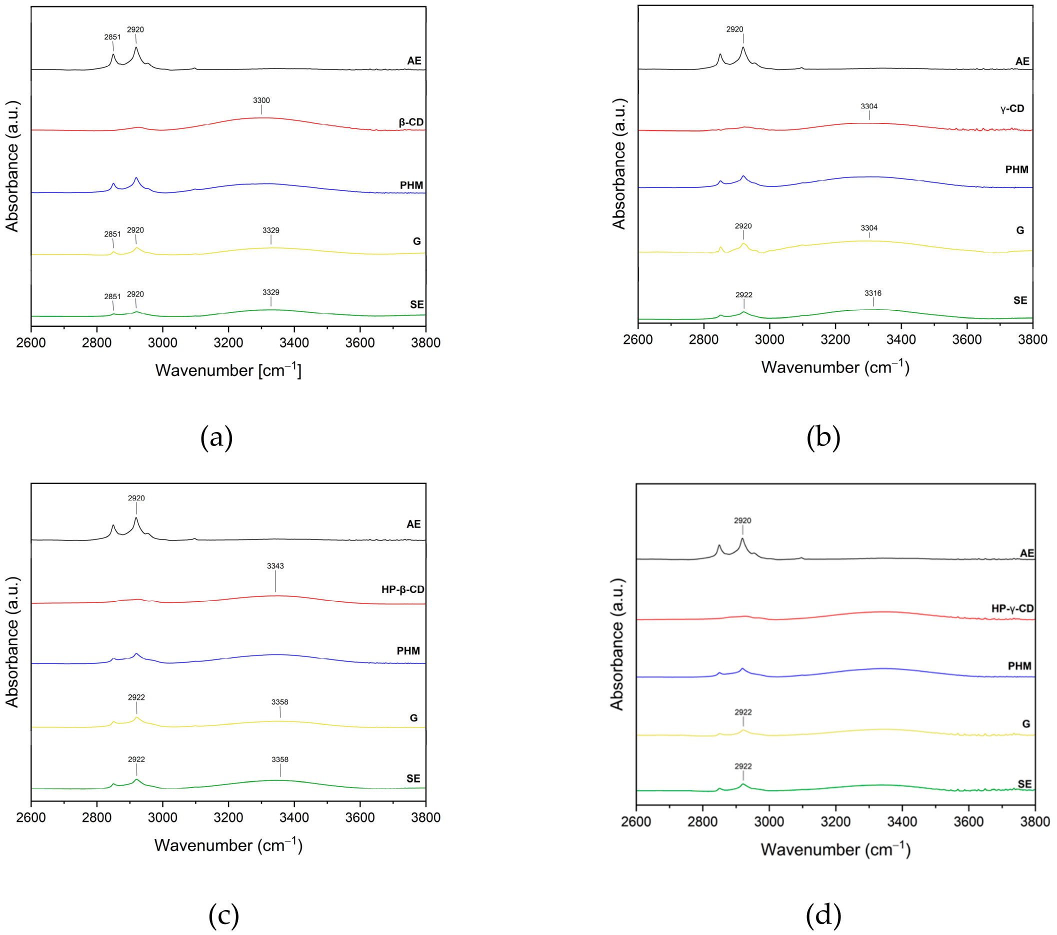

Figure S1. The results of FTIR analysis (2600-3800 cm^{-1}) of acetone extract (AE) (black), β-cyclodextrin (β-CD) (red), AE/β-CD 1:1.5 physical mixture (PHM) (blue), AE/β-CD 1:1.5 system by grinding (G) (yellow), and AE /β-CD 1:1.5 system by solvent evaporation (SE) (green) (a); The results of FTIR analysis (2600-3800 cm^{-1}) of acetone extract (AE) (black), γ- cyclodextrin (γ-CD) (red), AE/γ-CD 1:1.5 physical mixture (PHM) (blue), AE/γ-CD 1:1.5 system by grinding (G) (yellow), and AE/γ-CD 1:1.5 system by solvent evaporation (SE) (green) (b); The results of FTIR analysis (2600-3800 cm^{-1}) of acetone extract (AE) (black), 2-hydroxypropyl-β-cyclodextrin (HP-β-CD) (red), AE/HP-β-CD 1:1.5 physical mixture (PHM) (blue), AE/HP-β-CD 1:1.5 system by grinding (G) (yellow), and AE /HP-β-CD 1:1.5 system by solvent evaporation (SE) (green) (c); The results of FTIR analysis (2600-3800 cm^{-1}) of acetone extract (AE) (black), hydroxypropyl-γ-cyclodextrin (HP-γ-CD) (red), AE/HP-γ-CD 1:1.5 physical mixture (PHM) (blue), AE/HP-γ-CD 1:1.5 system by grinding (G) (yellow), and AE/HP-γ-CD 1:1.5 system by solvent evaporation (SE) (green) (d);

Table S2. Selected characteristic bands (in cm^{-1}) of acetone extract (AE), β-cyclodextrin (β-CD), and physical mixture (PHM) of AE and β-CD (PHM) for which changes were observed in AE/β-CD system prepared by grinding (G) and AE/β-CD system prepared by solvent evaporation (SE).

AE	β-CD	PHM	G	SE
**457**			*	*
**530**		+	↓	↓
	575		+	+
**598**		600 ↓	600 ↓	600 ↓
627		+	*	*
652		+	*	*
664		+	*	*
**679**		+	↓	*
**702**		+	704	704
	706	-	704	704
745		+	*	*
	754	-	756	756
**783**		783	781	783
**800**		+	↓	↓
**843**		845	845↓	843↓
**870**		868	868	864
	997	1003	1001	1001
	1018	1020	1022	1024
**1130**		+	↓	↓
**1207**		+	↓	↓
**1231**		↓	1233↓	1233↓
**1256**		↓	↓	↓
**1360**		↓	↓	↓
**1379**		+	↓	↓
**1414**		+	↓	*
1450		+	↓	↓
1576		+	↓	↓
**1614**		+	↓	*
1649		+	↓	↓
1695		+	↓	↓
2851		+	↓	↓
2920		+	↓	↓
	3300	+	3329	3329

Legend: bold value - peak corresponding to fumarprotocetraric acid (based on second derivative for the recorded FTIR spectra of AE), + - no shift of the peak location, ↓ decrease in intensity, * - disappearance of the peak.

Table S3. Selected characteristic bands (in cm-1) of acetone extract (AE), γ- cyclodextrin (γ-CD), and physical mixture (PHM) of AE and γ-CD for which changes were observed in AE/γ-CD system prepared by grinding (G) and AE/γ-CD-system prepared by solvent evaporation (SE).

AE	γ-CD	PHM	G	SE
	581	+	583	579
**598**		+	+	↓
**843**		+	+	845↓
**870**		+	+	866↓
	941	+	939	939
	997	+	999	999
	1018	+	1024	1020
**1207**		+	1209	1209
**1256**		+	1258	1258
1649		+	1651	1651
1695		+	1699	1699
1744		+	1746	1746
2920		+	+	2922

Legend: bold value - peak corresponding to fumarprotocetraric acid (based on second derivative for the recorded FTIR spectra of AE), + - no shift of the peak location.

Table S4. Selected characteristic bands (in cm-1) of acetone extract (AE), HP-β-CD-cyclodextrin (HP-β-CD), and physical mixture (PHM) of AE and HP-β-CD for which changes were observed in AE/HP-β-CD system prepared by grinding (G) and AE/ HP-β-CD-system prepared by solvent evaporation (SE).

AE	HP-β-CD	PHM	G	SE
**457**		↓	*	*
**760**		↓	*	*
	945	+	947	947
1005		+	1003↓	1003↓
**1207**		↓	1209	1209
**1231**		↓	1233↓	1233↓
**1256**		+	1258	1258
**1294**		+	1296	1296
**1315**		+	1317	1317
**1356**		+	+	1360
1649		+	1653	1653
1695		1697	1699	1699
1744		+	1746	1746
2920		+	2922	2922
	3343	+	3358	3358

Legend: bold value - peak corresponding to fumarprotocetraric acid (based on second derivative for the recorded FTIR spectra of AE), + - no shift of the peak location, ↓ - decrease in intensity, * - disappearance of the peak.

Table S5. Selected characteristic bands (in cm^{-1}) of acetone extract (AE), HP-γ-CD-cyclodextrin (HP-γ-CD), and physical mixture (PHM) of AE and HP-γ-CD for which changes were observed in AE/HP-γ-CD system prepared by grinding (G) and AE/HP-γ-CD-system prepared by solvent evaporation (SE).

AE	HP-γ-CD	PHM	G	SE
**598**		↓	↓	↓
**679**		↓	*	*
**702**		+↓	*	*
	706		704	704
	1018	+	1022	1022
**1130**		+	1128↓	1128↓
**1256**		1254	1258	+
**1294**		+	1296	1296
**1315**		+	1317	1317
1649		+	1653	1653
1695		+	1701	1701
1744		+	1746	1746
2920		+	2922	2922

Legend: bold value - peak corresponding to fumarprotocetraric acid (based on second derivative for the recorded FTIR spectra of AE), + - no shift of the peak location, ↓ - decrease in intensity, * - disappearance of the peak.

Table S6. Selected characteristic bands (in cm^{-1}) of methanol extract (ME), β-cyclodextrin (β-CD), and physical mixture (PHM) of ME and β-CD for which changes were observed in ME/β-CD system prepared by grinding (G) and ME/β-CD system prepared by solvent evaporation (SE).

ME	β-CD	PHM	G	SE
**494**		+	*	*
	858	-	+	+
	997	+	999	999
1022				
	1018	+	1022	1022
**1256**		+	1258	*
2851		+	*	*
	3300	+	3291	3296

Legend: bold value - peak corresponding to fumarprotocetraric acid (based on second derivative for the recorded FTIR spectra of ME), + - no shift of the peak location, * - disappearance of the peak.

Table S7. Selected characteristic bands (in cm^{-1}) of methanol extract (ME), γ-CD-cyclodextrin (γ-CD), and physical mixture (PHM) of ME and γ-CD for which changes were observed in ME/γ-CD system prepared by grinding (G) and ME/γ-CD-system prepared by solvent evaporation (SE)

ME	γ-CD	PHM	G	SE
	581	+	575	573
**700**		704	702	702
	941	+	937	935
	997	+	+	1001
	1018	+	1022	1020
2920		2922	2920	2918
	3304	+	3298	3294

Legend: bold value - peak corresponding to fumarprotocetraric acid (based on second derivative for the recorded FTIR spectra of ME), + - no shift of the peak location.

Table S8. Selected characteristic bands (in cm^{-1}) of methanol extract (ME), HP-β-CD-cyclodextrin (HP-β-CD), and physical mixture (PHM) of ME and HP-β-CD for which changes were observed in ME/HP-β-CD system prepared by grinding (G) and ME/HP-β-CD-system prepared by solvent evaporation (SE).

ME	HP-β-CD	PHM	G	SE
	851	+	854	851
	945	+	941	947
1022		-	1018	-
1153		-	1152	1152
	3343	+	3312	3337

Legend: + - no shift of the peak location.

Table S9. Selected characteristic bands (in cm^{-1}) of methanol extract (ME), HP-γ-CD-cyclodextrin (HP-γ-CD), and physical mixture of ME and HP-γ-CD (PHM) for which changes were observed in ME/HP-γ-CD system prepared by grinding (G) and ME/HP-γ-CD-system prepared by solvent evaporation (SE).

ME	HP-γ-CD	PHM	G	SE
	941	+	943	945
	1018	+	1003	1003
	1150	+	1153	1155
	2928	+	-	-
2920		-	2922	2922
	3360	+	3335	3329

Legend: ME - methanol extract, + - no shift of the peak location, ↓ - decrease in intensity, * - disappearance of the peak.

Table S10. HPLC method validation parameters

Detection wavelength [nm]	254
Linerity a ± Sa<br>b ± Sb	a = 177.84 ± 1.5857<br>b - insignificant
Correlation coefficient (r)	R = 0.9998
Linearity range [ug]	0.02 – 0.8 (10-100 µg/ml)
Limit of detection [ug] (LOD)	0.02026
Limit of quantification [ug] (LOQ)	0.06140

Sa – standard devioation, Sb – Standard devioation of the intersection point, t calculated values of the Student's t-test. Tα f = 2.447. Critical values of the Student's test for degrees of freedom f = 6 significance level α = 0.05
